# Regulation of the Type I-F CRISPR-Cas system by CRP-cAMP and GalM controls spacer acquisition and interference

**DOI:** 10.1093/nar/gkv517

**Published:** 2015-05-24

**Authors:** Adrian G. Patterson, James T. Chang, Corinda Taylor, Peter C. Fineran

**Affiliations:** Department of Microbiology and Immunology, University of Otago, PO Box 56, Dunedin 9054, New Zealand

## Abstract

The CRISPR-Cas prokaryotic ‘adaptive immune systems’ represent a sophisticated defence strategy providing bacteria and archaea with protection from invading genetic elements, such as bacteriophages or plasmids. Despite intensive research into their mechanism and application, how CRISPR-Cas systems are regulated is less clear, and nothing is known about the regulation of Type I-F systems. We used *Pectobacterium atrosepticum*, a Gram-negative phytopathogen, to study CRISPR-Cas regulation, since it contains a single Type I-F system. The CRP-cAMP complex activated the *cas* operon, increasing the expression of the adaptation genes *cas1* and *cas2–3* in addition to the genes encoding the Csy surveillance complex. Mutation of *crp* or *cyaA* (encoding adenylate cyclase) resulted in reductions in both primed spacer acquisition and interference. Furthermore, we identified a galactose mutarotase, GalM, which reduced *cas* operon expression in a CRP- and CyaA-dependent manner. We propose that the Type I-F system senses metabolic changes, such as sugar availability, and regulates *cas* genes to initiate an appropriate defence response. Indeed, elevated glucose levels reduced *cas* expression in a CRP- and CyaA-dependent manner. Taken together, these findings highlight that a metabolite-sensing regulatory pathway controls expression of the Type I-F CRISPR-Cas system to modulate levels of adaptation and interference.

## INTRODUCTION

Bacteria and archaea are regularly subjected to invasion by foreign nucleic acids and, as such, experience a selective pressure that has favoured the development of multiple defence mechanisms. Genetic loci known as clustered regularly interspaced short palindromic repeats (CRISPR) (1,2) and their CRISPR-associated (Cas) proteins (3) facilitate the targeted degradation of horizontally acquired genetic elements such as bacteriophages (4) and plasmids (5) (for reviews see (6–9)). The immunity provided by these systems relies on the acquisition of short invader-derived nucleotide sequences, termed ‘spacers’, which are incorporated between direct repeats in the CRISPR arrays. In doing so, the cell essentially forms a ‘genetic memory’ of previous exposures to foreign elements. The CRISPR-Cas mechanism is generally divided into three separate phases. Firstly, during an ‘adaptation’ or ‘acquisition’ phase, a target sequence within the foreign element (protospacer) is recognised by Cas proteins and incorporated as a new spacer into a CRISPR array, resulting in repeat duplication. This process of acquiring genetic information from an invader that has not previously been encountered is termed ‘naïve’ acquisition (10). Secondly, initiation of transcription from a leader sequence preceding the CRISPR array results in the formation of pre-CRISPR-RNA (crRNA), which is subsequently cleaved at direct repeats flanking the spacer sequences to yield mature crRNAs with a sequence complementary to that of the invading element. Finally, these short crRNAs then interact with various Cas proteins, forming ribonucleoprotein complexes, which mediate the destruction of invading DNA in an interference process reliant on base pairing with the protospacer.

The heritable genetic memory provided by CRISPR-Cas systems provides an immediate response to successive viral or plasmid invasions. However, interference may be avoided by escape mutations in the invader that impairs interference (4,11,12). In many CRISPR-Cas systems a short sequence adjacent to the protospacer (termed a protospacer adjacent motif (PAM)) is also necessary for interference (11,13,14). To overcome escape mutations in the protospacer or PAM, CRISPR-Cas immunity can be bolstered via a phenomenon known as ‘primed acquisition’ (15,16). During this process, a crRNA, derived from the pre-existing spacer, guides the surveillance complex to the target with the escape mutation, yet cannot elicit interference. For example, a single nucleotide mutation in either the PAM or an ∼8 nucleotide PAM-proximal seed region is sufficient to limit interference but stimulate priming (12,15). Instead of immediate interference, the adaptation machinery samples additional spacers, which are incorporated into the CRISPR array. The exact mechanism for primed acquisition is yet to be elucidated; however, recent evidence in a Type I-F system supports a localised translocation model in which new spacers are acquired preferentially around the primed protospacer (17). By acquiring additional functional spacers, the bacterium is able to more efficiently identify the invading genetic element and limits the success of mutants that otherwise would escape detection.

CRISPR-Cas systems are found in a range of bacteria and archaea, with CRISPRdb indicating CRISPR arrays are present in approximately 45% and 84% of genomes, respectively (18). Three major groups of CRISPR-Cas systems (Types I–III) have been identified, in addition to various subtypes, each categorised by the unique profile of *cas* genes accompanying the CRISPR array(s) (7). Given the far-reaching biotechnological applications of CRISPR-Cas systems, investigation into their mechanism has been a major focus in recent years, specifically crRNA biogenesis and interference (19). In contrast, there is a paucity of information regarding the regulation of these systems, with most work focusing on the Type I-E system in *Escherichia coli*. Of the limited literature available, there are varied forms of control across different subtypes. Such variation is highlighted when considering control by the cAMP receptor protein (CRP), which has been demonstrated to have contrasting roles across several CRISPR-Cas systems. In the Type I-E and III-A subtypes of *Thermus thermophilus*, CRP functions as an activator (20,21), but in *E. coli*, CRP represses the Type I-E system (22). In *E. coli*, H-NS functions as another repressor which influences both CRISPR and *cas* promoters and its deletion results in enhanced phage resistance (23,24). The leucine-responsive regulatory protein (LRP) represses the Type I-E system in *Salmonella enterica* serovar Typhi, but has no detectable role in *E. coli* CRISPR-Cas regulation (23,25). Consequently, rather than a universal mode of regulation, it seems control of expression is tailored across different subtypes and species. This variation might reflect the diversity of CRISPR-Cas systems and the different conditions and frequency with which bacteria encounter horizontally acquired genetic elements.

**Figure 1.**
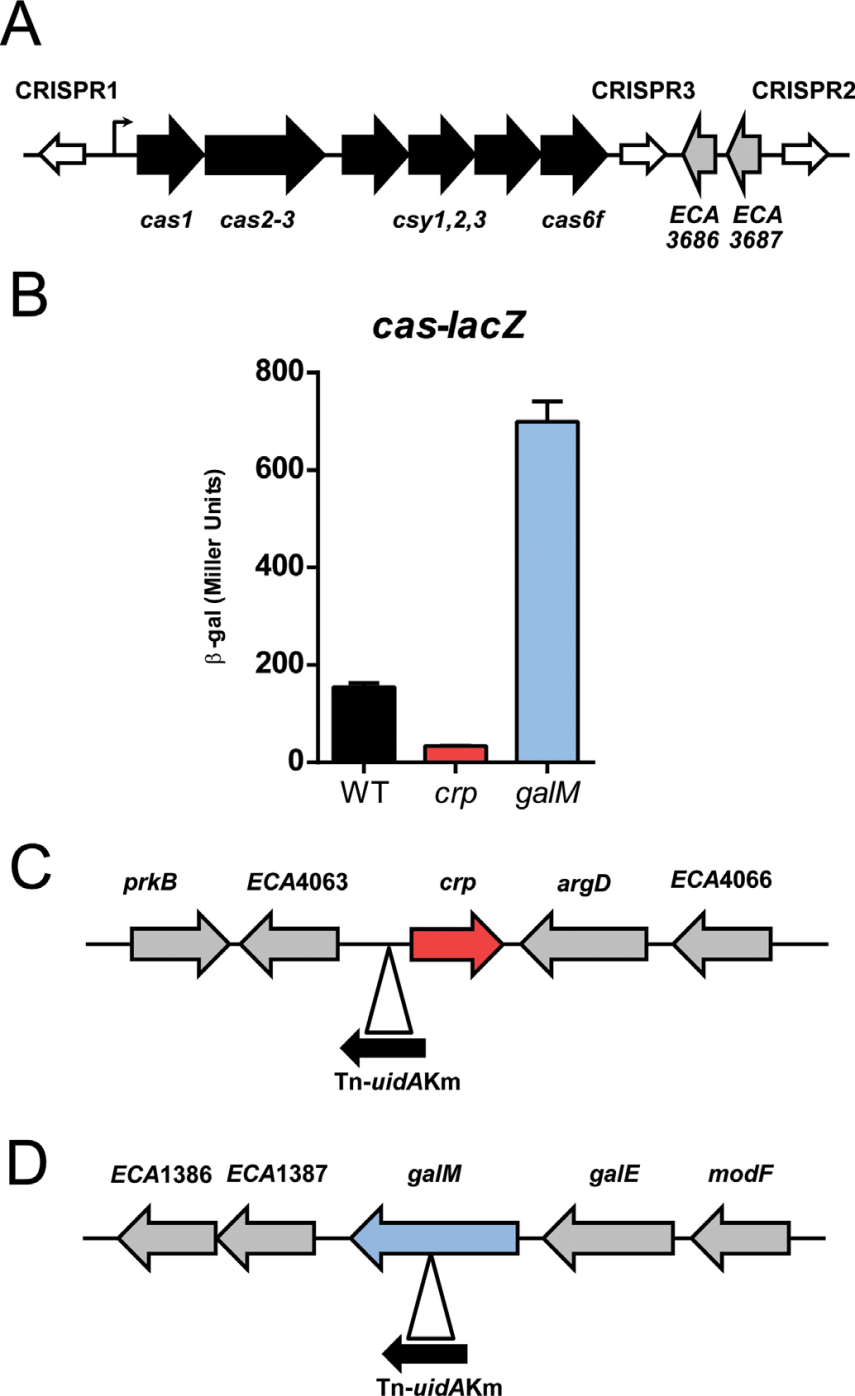
Transposon insertions in *crp* and *galM* differentially affect *cas* expression. (**A**) Schematic of the *P. atrosepticum* Type I-F CRISPR-Cas system. The system contains 6 genes linked in an operon (black) consisting of *cas1, cas2–3, csy1, csy2, csy3* and *cas6f*, with transcription being initiated from the *cas1* promoter (for details see Figure 3B). Three separate CRISPR arrays (white) are also present within the genome, named CRISPR1–3, with 28, 10 and 3 spacers, respectively. (**B**) Activity of a *cas-lacZ* transcriptional/translational fusion in the WT (PCF79, black), *crp* mutant (PCF173, red) and *galM* mutant (PCF85, blue). The mutants were obtained by transposon mutagenesis of strain PCF79 and β-gal activity was measured after 12 h growth (see Materials and Methods). Data shown are the mean ± SD (*n* = 3). Location and orientation of transposon (black arrow) in the genomes of the (**C**) *crp* mutant (inserted at 4529114 bp) or (**D**) *galM* mutant (inserted at 1571635 bp).

*Pectobacterium atrosepticum* (formerly *Erwinia carotovora* subsp. *atroseptica*) is a Gram-negative phytopathogen which harbours three CRISPR arrays (CRISPR1–3) and a single set of Type I-F *cas* genes including *cas1, cas2–3, csy1, csy2, csy3* and *cas6f* (*csy4*) (Figure 1A) (26,27). These *cas* genes are expressed as a single operon during mid-exponential growth, with transcription being initiated from the *cas1* promoter (27,28). The apparent disparity involved in regulatory control of different CRISPR-Cas systems means that the presence of a single subtype in *P. atrosepticum* makes it an attractive model to study control of Type I-F CRISPR-Cas systems. Our findings demonstrate that the CRP-cAMP complex activates the *cas* operon, while a previously unreported gene, *galM*, reduces *cas* expression. Furthermore, glucose availability repressed *cas* operon transcription. The altered *cas* expression profiles in all mutants correlated with changes in CRISPR-Cas-mediated interference as well as the primed acquisition of new spacers into the genomic CRISPR loci. Our findings reveal that carbon (glucose) availability can be altered by GalM and sensed by CyaA and CRP to control *cas* expression, and ultimately, CRISPR-Cas adaptation and interference.

## MATERIALS AND METHODS

### Bacterial strains and growth conditions

Strains and plasmids used in this study are given in Supplementary Tables S1 and S2. Details of their construction are provided in Supplementary Materials and Methods. *P. atrosepticum* was grown at 25°C and *E. coli* at 37°C in either Lysogeny Broth (LB) at 180 rpm or on LB-agar (LBA) plates containing 1.5% (w v^−1^) agar. Minimal media contained 40 mM K_2_HPO_4_, 14.6 mM KH_2_PO_4_, 0.4 mM MgSO_4_, 7.6 mM (NH_4_)_2_SO_4_ and 0.2% (w v^−1^) or 2% (w v^−1^) carbon source. When required, media were supplemented with ampicillin (Ap; 100 μg ml^−1^), chloramphenicol (Cm; 25 μg ml^−1^), kanamycin (Km; 50 μg ml^−1^), tetracycline (Tc; 10 μg ml^−1^) or D-glucose (2% w v^-1^). Growth was measured in a Jenway 6300 Spectrophotometer at 600 nm (OD_600_) when grown in flasks or in a Modulus Microplate Multimode Reader with a 9300–050 Absorbance Module at 600 nm in 96-well microtitre plates. All experiments were repeated in at least three biological replicates.

### Transposon mutagenesis

One ml of overnight cultures of the donor (*E. coli* BW20767 (pKRCPN2)) and the recipient (*P. atrosepticum* PCF79; *cas-lacZ*) strains were pelleted at 3030 ×*g* for 5 min in a microcentrifuge and the pellet re-suspended in 1 ml LB. This step was repeated twice to remove antibiotics. Twenty μl of each the donor and recipient were mixed and spotted onto LBA and left overnight to allow conjugation of pKRCPN2 which harbours the miniTn*5*-based Tn-DS1028*uidA*Km transposon (Kevin Roberts, unpublished). Each resultant mating patch was scraped off, re-suspended in 1 ml LB, diluted 100-fold and 100 μl aliquots were plated on LBA containing Cm, Km and 20 μg ml^−1^ X-gal (5-bromo-4-chloro-3-indoyl-D-galactopyranoside). Plates were incubated for 2 d and mutants displaying altered colony colour on X-gal compared with PCF79 were isolated. Approximately 32 000 mutants were visually screened and ∼230 were isolated and streaked out to generate pure isolates. These mutants were grown in 10 ml of LB overnight for 12 h and quantitatively assayed using β-galactosidase (β-gal) assays as described previously (27). Mutants with the lowest *lacZ* activity and one with the highest activity were analysed further. The insertion sites were mapped by arbitrary polymerase chain reaction (PCR) as described previously (29) with the transposon specific primers PF294, PF337, PF338 and PF1209. All primers used in this study are provided in Supplementary Table S3. Overnight cultures of the *crp* and *galM* transposon mutants were diluted to an OD_600_ of 0.04 in 25 ml LB in 250 ml flasks and incubated at 200 rpm. The β-gal activity was assessed after 12 h growth (27).

### Plasmid loss and spacer acquisition assays

Plasmids pPF571 (non-targeted ‘naïve’ control) and pPF574 (priming vector containing the protospacer targeted by spacer 1 from CRISPR1, but with a non-consensus PAM to initiate priming) (17) were transformed into electrocompetent WT (REM200), *crp* (PCF112), *cyaA* (PCF113) and *galM* (PCF117) using 0.1 cm electroporation cuvettes in a BioRad ‘Gene Pulser’ set to 200 Ω, 25 μFD and 1.8 kV and plated directly on LBA with Tc (28). Overnight cultures of each transformant were set up in 5 ml of LB without antibiotics and passaged for 5 d by transfer of 10 μl to 5 ml of fresh LB. Additionally, −80°C stocks were prepared daily, by adding 500 μl of culture to 500 μl of 50% glycerol, and a 10^−6^ dilution plated on LBA containing 1 mM IPTG to induce the plasmid-encoded mCherry. After incubation at 25°C, white colonies (indicating plasmid loss) were counted and pooled genomic DNA from glycerol stocks was screened via PCR using primers PF1461 + PF1470 for CRISPR1, PF1464 + PF1473 for CRISPR2 and PF1467 + PF1476 for CRISPR3. PCR products were separated by 3% agarose gel electrophoresis to detect expansion of CRISPR loci caused by spacer incorporation.

### Conjugation efficiency assay

*E. coli* S-17 λ*pir* were used as donor cells for the conjugation of control (pPF571) and CRISPR-Cas-targeted (pPF572; contains a protospacer targeted by spacer 1 from CRISPR1) plasmids to WT (REM200), *crp* (PCF112), *cyaA* (PCF113) and *galM* (PCF117) recipients (17). The pPF572 plasmid was constructed using PF1365 (contains protospacer sequence and consensus GG PAM) and PF210 to amplify the Tc resistance cassette from pTRB31 and the resulting amplicon was ligated into pQE-80L-oriT-mCherry digested with XhoI and BspHI (compatible ends with NcoI site on PF1365). Donors and recipients were grown overnight in LB with the appropriate antibiotics, the OD_600_ adjusted to 1 and cells washed twice with LB. The donors and recipients were mixed (1:1 ratio), 5 μl of the mixture spotted on 0.2 μm filters (Millipore) on LBA and incubated for 24 h. Cells were resuspended in 2 ml phosphate buffered saline (PBS) by vortexing the filters and dilution series were plated on either glucose (0.2%) minimal medium (recipients) or glucose (0.2%) minimal medium with Tc (transconjugants). The efficiency of conjugation was calculated as transconjugants per recipients.

### β-galactosidase assay

All integrative *lacZ* reporter strains were grown in 1 ml of LB with Tc within individual wells of labcon deep-96 square well plate. Plates were incubated with 12 000 rpm shaking at 25°C using a BioProducts incumix microplate shaker. Expression analysis was performed using the fluorogenic substrate of β-galactosidase: 4-Methylumbelliferyl β-D-galactoside (MUG) (30). Samples of 100 μl were extracted at specific time points and frozen in separate 96-well microtitre plates at −80°C. Ten μl volumes of each sample were subsequently frozen at −80°C immediately prior to the assay and thawed for 10 min at 37°C. During this time the final reaction buffer (PBS, 2 mg ml^−1^ lysozyme, 250 μg ml^−1^ MUG) was prepared, from which 100 μl was added to the thawed samples. The relative change in fluorescence was immediately monitored using a Varioskan Flash Multimode Reader (Thermo Fisher Scientific) according to the following parameters: excitation 365 nm, emission 455 nm, 37°C, 8 reads per well, measured every 1 min for 30 min. Relative fluorescent units (RFUs) per minute (min^−1^) were calculated using the linear increase in fluorescence which was normalised to the OD_600_ of the sample (RFU/sec/OD_600_).

## RESULTS

### Mutations in *crp* and *galM* affect *cas* expression

To identify regulators of the Type I-F CRISPR-Cas system (Figure 1A), a transposon mutagenesis was performed using a *P. atrosepticum* reporter strain containing an in-frame chromosomal transcriptional/translational *lacZ* fusion to the *cas* operon (*cas1*) promoter. In this strain, *cas* expression increases throughout growth, rising from mid-exponential phase and continuing to increase in late exponential/stationary phase (27,28). Following an initial visual plate screen, transposon mutants were quantified for *cas1* expression and those showing the highest and lowest β-galactosidase activity were sequenced. Transposon insertions that abolished or greatly reduced *lacZ* activity were mapped to either the *lacZ* gene (false negatives) or upstream of a gene with 99% nucleotide similarity to *E. coli crp* (Figure 1B and C). Another transposon was mapped to the open reading frame of the *P. atrosepticum galM* gene and this mutant exhibited a strong increase in *cas*-*lacZ* expression (Figure 1B and D). Therefore, transposon insertions upstream of *crp* and within *galM* have opposing effects on *cas* expression.

### CRP-cAMP activates *cas* operon expression

**Figure 2.**
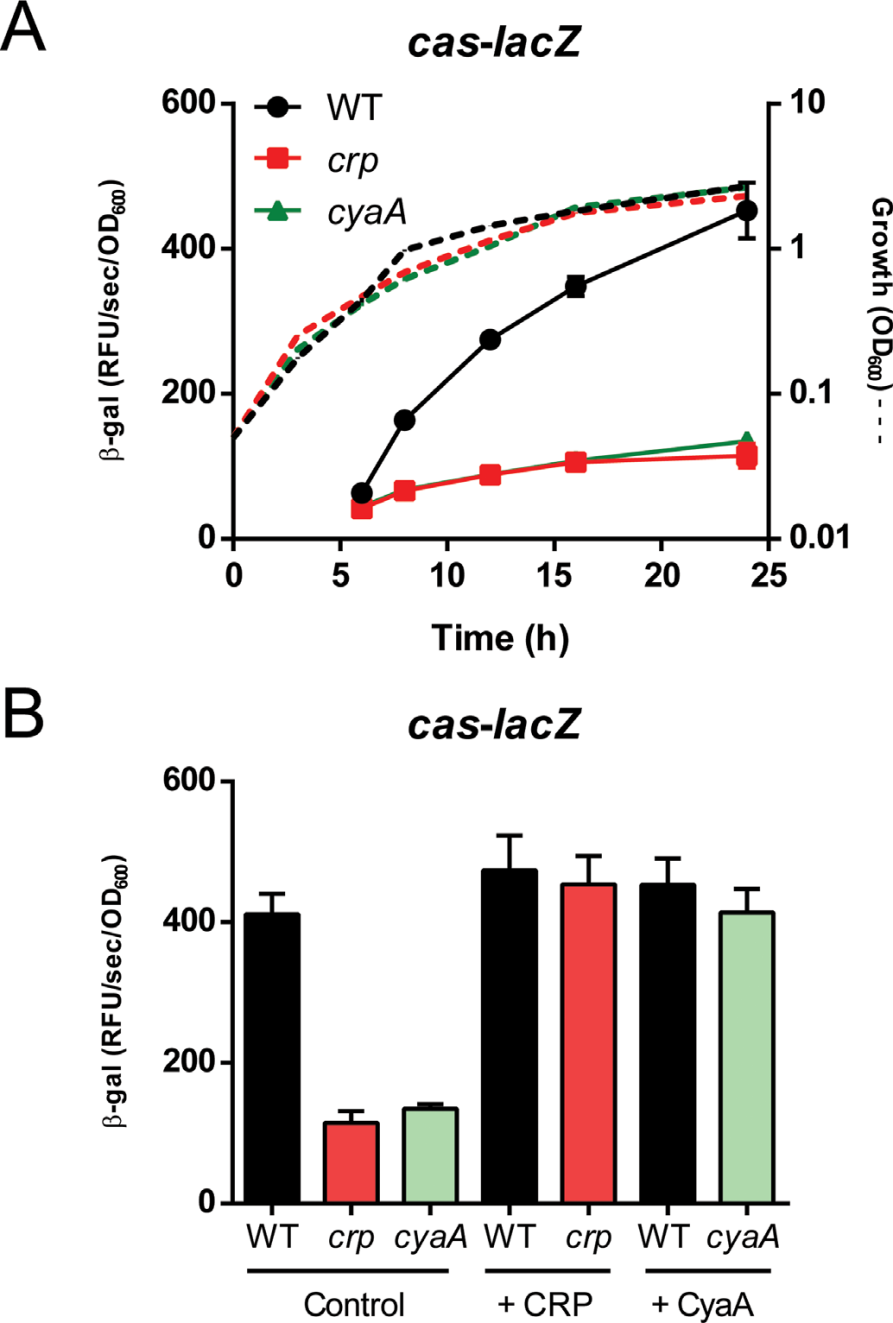
CRP-cAMP activates *cas* operon expression. (**A**) Expression of the integrative *cas-lacZ* promoter reporter in the WT (PCF123, black), *crp* mutant (PCF124, red) or *cyaA* mutant (PCF125, green) (see Materials and Methods). Dashed lines represent growth in LB and solid lines represent *cas1* promoter activity. (**B**) Complementation analysis using an empty control vector (pQE-80L), CRP (pPF600) or CyaA (pPF622) within the backgrounds in (A) 24 h post inoculation with 0.1 mM IPTG induction. Data shown are the mean ± SD (*n* = 3).

The *crp* gene encodes a transcription factor, cAMP receptor protein (CRP), responsible for the regulation of a wide range of promoters (31). To modulate expression, CRP forms a dimeric complex and binds cAMP, which is produced by adenylate cyclase (CyaA) (32–34). As the transposon mapped within the *crp* promoter, we deleted the entire *crp* gene to ensure effects on *cas* expression were due to loss of CRP. To confirm that CRP was a regulator of the *cas* operon, expression from a single copy integrative *cas*-*lacZ* reporter was assessed (Supplementary Figure S1). In the absence of *crp, cas1* activity was reduced almost 4-fold compared with the WT, indicating that CRP activates *cas1* promoter expression (Figure 2A). The dependency of *cas* operon expression on the presence of cAMP was assessed by generating a strain with a deletion of *cyaA* replaced by a chloramphenicol resistance cassette. In the *cyaA* mutant, *cas1* promoter activity was reduced compared with the WT and the level was indistinguishable to that of the *crp* mutant (Figure 2A). Both the *crp* and *cyaA* strains exhibited a minor growth defect compared with the WT strain (Figure 2A). However, this was not sufficient to account for the difference in expression. In addition, *cas1* expression was similarly reduced in a *crp cyaA* double mutant (Supplementary Figure S2). Therefore, there was no additive effect for the double mutant, presumably due to the dual requirement of *cyaA* and *crp* for *cas1* activation. Introduction of *crp* and *cyaA* on plasmids restored expression to WT levels in the *crp* and *cyaA* mutant backgrounds, respectively (Figure 2B). Therefore, the CRP-cAMP complex is responsible for activation of the Type I-F *cas* operon in *P. atrosepticum*.

### A CRP binding site in the *cas1* promoter is required for activation

**Figure 3.**
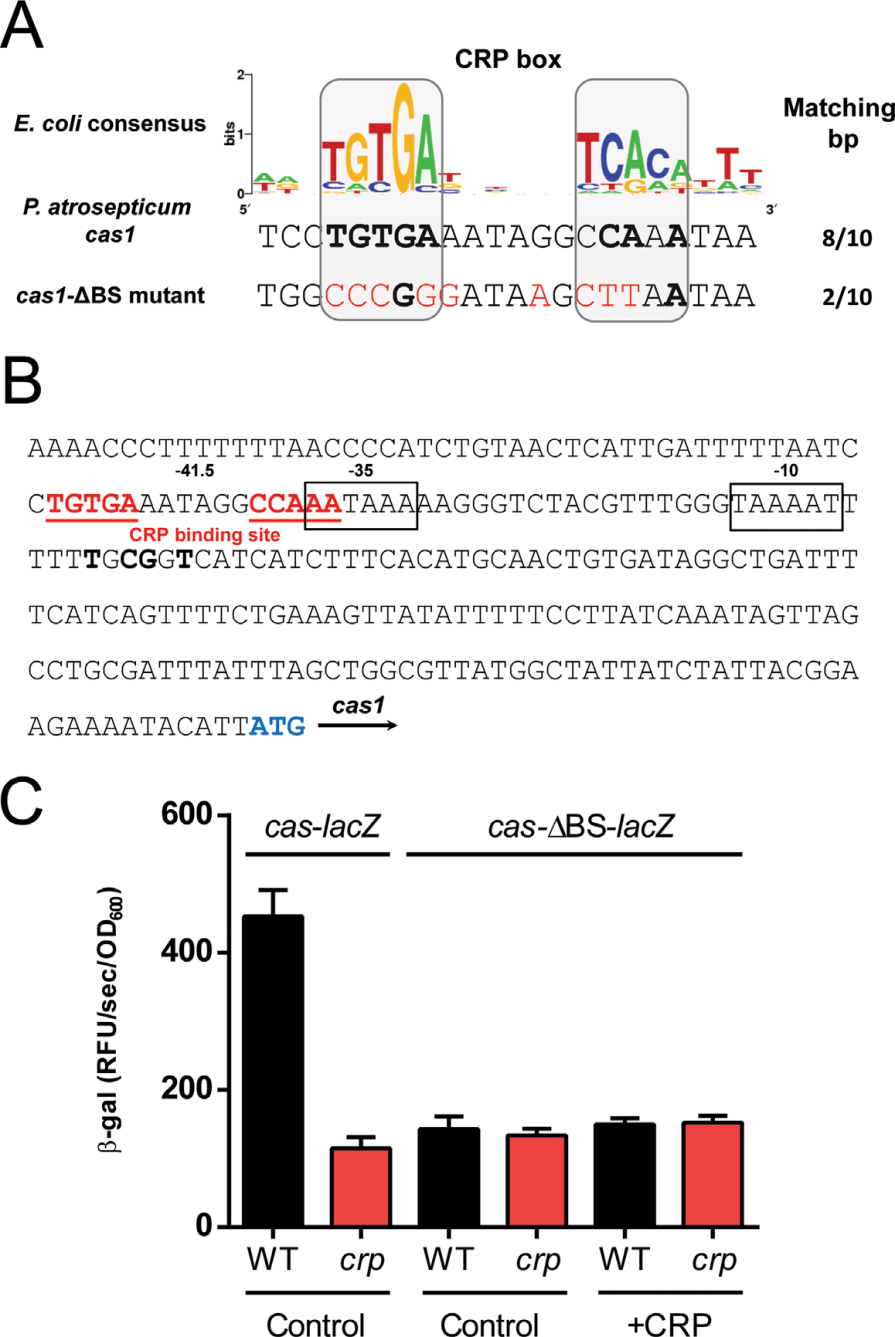
A CRP binding site in the *cas1* promoter is required for activation. (**A**) The *P. atrosepticum cas1* promoter (middle) contains a CRP-box similar to the CRP-binding consensus found in *E. coli* (top) (data from Zheng *et al*. (2004) (47)). An altered *cas1* CRP-box was generated to investigate CRP binding (bottom). Bases matching the consensus are bold and mutated bases are shown in red. (**B**) The putative CRP-box (red) is located ∼200 bp upstream of the *cas1* start codon and overlaps the −35 site, centred at −41.5. Transcriptional start sites predicted by 5′ RACE are shown in bold (27). (**C**) Expression of the *cas1* promoter or the *cas1* promoter containing mutated binding site (*cas*-ΔBS) in the WT (black) or *crp* mutant (red) was measured 24 h post inoculation (using the integrative *cas*-*lacZ* reporters on pPF705 and pPF706, respectively). CRP was also expressed (pPF600) in the *cas*-ΔBS backgrounds compared with an empty control vector (pQE-80L) with 0.1 mM IPTG (compare with Figure 2B). Data shown are the mean ± SD (*n* = 3).

For CRP to modulate gene expression, the CRP-cAMP complex must bind to a specific sequence within the promoter region located either upstream of core promoter elements (Class I), at a site overlapping the −35 element (Class II), or via interactions with alternative co-regulators (Class III) (35,36). Analysis of the *cas1* promoter revealed a putative CRP-binding site (TGTGA-N_6_-CCAAA) that shared 8 of 10 bp (base pairs) with the *E. coli* consensus (TGTGA-N_6_-TCACA) (Figure 3A). This predicted CRP-box overlapped the −35 site in the *cas1* promoter and was centred exactly at −41.5 (Figure 3B). This location suggests that CRP-mediated regulation of *cas1* occurs via a Class II mechanism, involving interactions with both the C- and N-terminal domains of the RNA polymerase (RNAP) alpha subunit (37). To evaluate whether the predicted CRP-box was required for CRP-cAMP activation of *cas1*, it was mutated without altering the −35 sequence (Figure 3A). In the WT background, expression of the *cas1* promoter with the mutant CRP-box was reduced compared with a reporter possessing the intact CRP-box (Figure 3C). To examine if CRP activates *cas* expression via this binding site, expression of the reporter was measured in the *crp* deletion strain. Deletion of *crp* did not further decrease *cas* promoter activity when the binding site was mutated (Figure 3C). In addition, in the CRP-box mutant, *cas* expression could not be restored when functional CRP was re-introduced (Figure 3C). These results demonstrate that the CRP-cAMP complex requires this specific CRP-box to activate *cas* operon expression.

### GalM activity reduces *cas* operon expression

**Figure 4.**
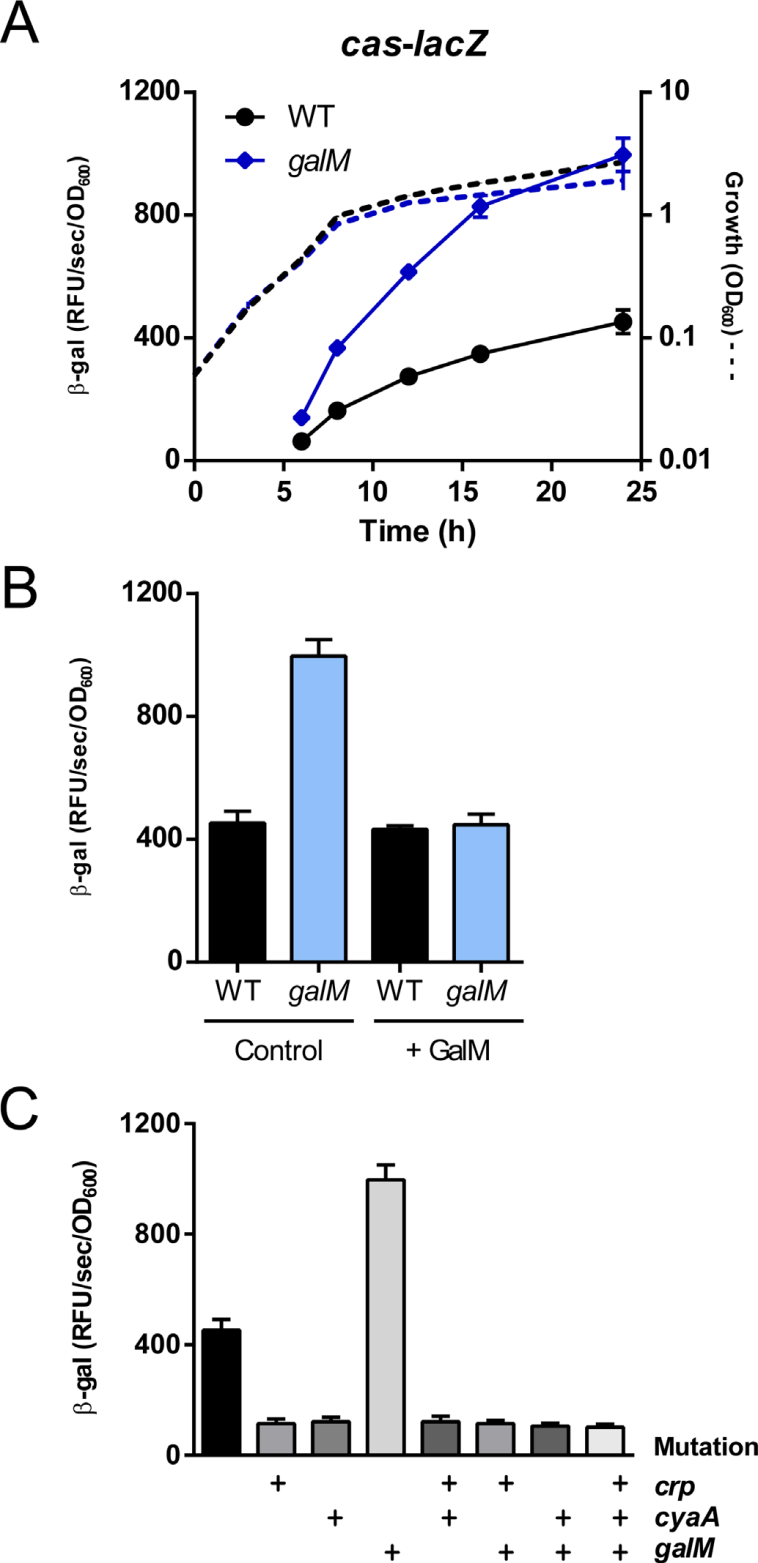
GalM activity reduces *cas* operon expression. (**A**) Expression of the integrative *cas-lacZ* promoter reporter in the WT (PCF123, black) or *galM* mutant (PCF126, blue) (see Materials and Methods). Dashed lines represent growth in LB and solid lines represent *cas1* expression. (**B**) Complementation analysis using either a control vector (pQE-80L-oriT) or GalM (pPF620) within each background 24 h post inoculation with 0.1 mM IPTG induction. (**C**) Expression of the *cas1* promoter in the WT (PF123), *crp* (PCF124), *cyaA* (PCF125), *galM* (PCF126), *crp cyaA* (PCF127), *crp galM* (PCF128), *cyaA galM* (PCF129) or *crp cyaA galM* (PCF130) backgrounds at 24 h post inoculation. The ‘+’ symbol denotes the presence of the *crp, cyaA or galM* mutations. Data shown are the mean ± SD (*n* = 3).

The other putative regulator identified in the transposon screen showed similarity to *E. coli galM*, which encodes a galactose mutarotase responsible for the epimerisation of β-D-galactose into α-D-galactose. Mutation of *galM* resulted in a >2-fold increase in *cas1* expression compared with the WT, demonstrating that GalM reduces transcription from the *cas1* promoter (Figure 4A). Despite the *galM* mutant exhibiting a minor growth defect compared with the WT strain, this did not account for the difference in expression observed. The increase in expression was complemented when GalM was produced from an inducible plasmid, whereas GalM overexpression had no effect on normal *cas1* expression in the WT (Figure 4B). Since GalM does not contain any recognisable DNA-binding domains, it is likely to be indirectly controlling *cas1* expression. Based on the role of this protein in other bacteria, it was hypothesised that GalM increases intracellular glucose, which in turn limits adenylate cyclase (CyaA) activity and CRP-cAMP-dependent activation of the *cas1* promoter (38–43). To assess if GalM was functioning via the CRP-cAMP complex, *crp, galM* and *cyaA, galM* double mutants and a *crp, cyaA, galM* triple mutant were generated and expression of the *cas1* reporter was quantified. For each strain, the increase in *cas1* expression in the *galM* mutant was abolished by deletion of *crp* or *cyaA* (Figure 4C). This is consistent with GalM exerting an ‘anti-activator’ effect by increasing glucose which reduces cAMP production by CyaA. We postulate that GalM activity, through decreased availability of the CRP-cAMP complex, restricts the expression of *cas1*.

### Glucose abundance represses the *cas* operon

**Figure 5.**
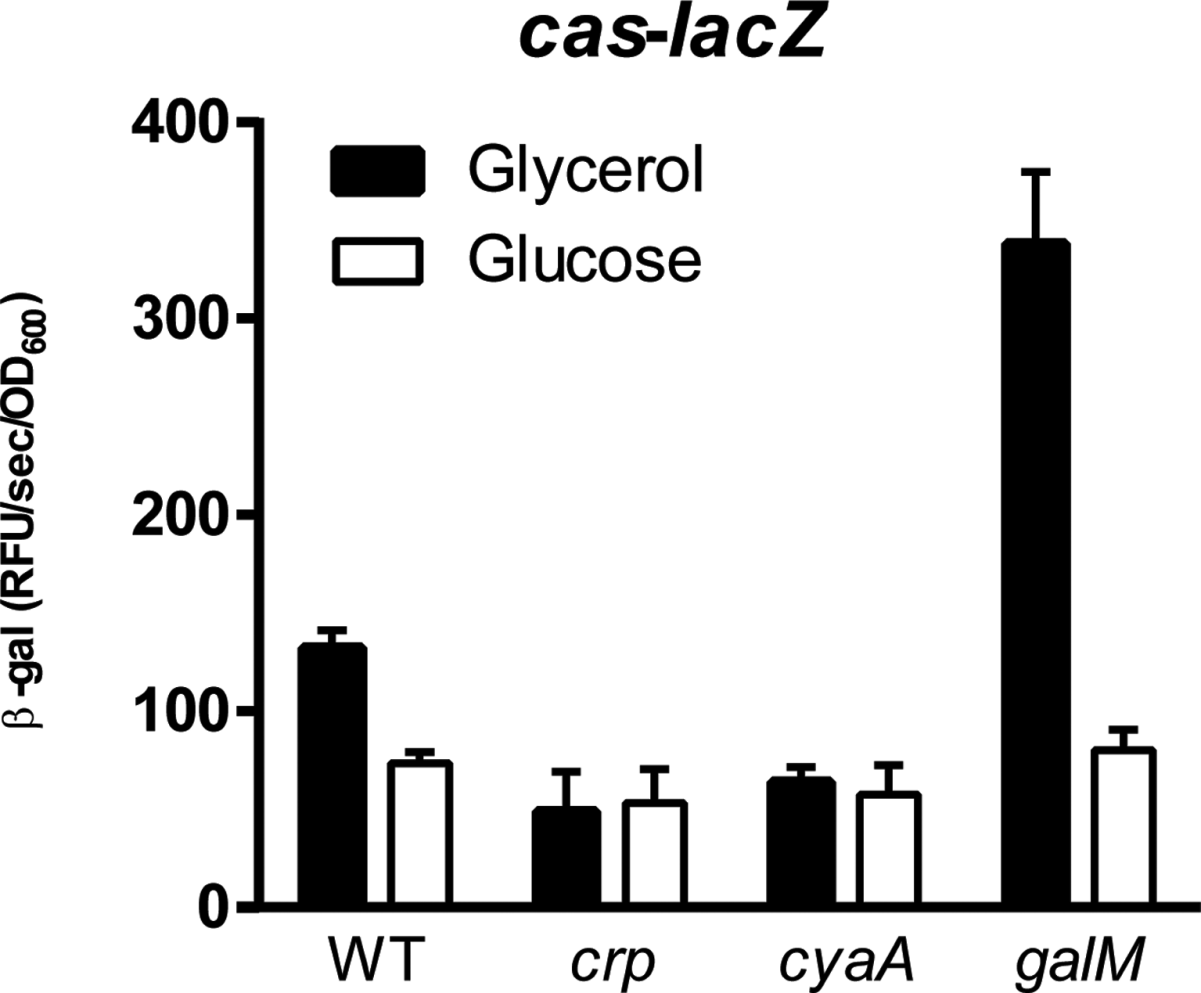
Glucose abundance influences *cas* expression. Expression of the integrative *cas-lacZ* promoter reporter in the WT (PCF123), *crp* mutant (PCF124), *cyaA* mutant (PCF125) or *galM* mutant (PCF126) when grown in minimal medium with either glycerol or glucose (2% w v^−1^) as the sole carbon source at 24 h (see Materials and Methods). Data shown are the mean ± SD (*n* = 3).

To directly investigate the influence of glucose on *cas* expression, various strains were grown in defined medium supplemented with either glucose or glycerol as the carbon source. Glycerol was selected as a control as it feeds into the glycolytic pathway and consequently should not result in the upregulation of genes involved in glucose metabolism (44). In the WT, *cas* expression was reduced by glucose compared with glycerol (Figure 5). As expected, *cas* expression in the *crp* or *cyaA* backgrounds was unresponsive to glucose (Figure 5). The *galM* mutant exhibited increased *cas* expression in glycerol, which was reduced to levels comparable to the *crp* and *cyaA* mutants when grown with glucose (Figure 5). As expected, the predicted reduction in available glucose in the *galM* mutant, which led to an increase in *cas* expression, could be reversed with excess glucose. Glucose levels (and GalM) therefore influence *cas* expression via limitation of adenylate cyclase activity and CRP activation.

### Altered *cas* expression influences adaptation

**Figure 6.**
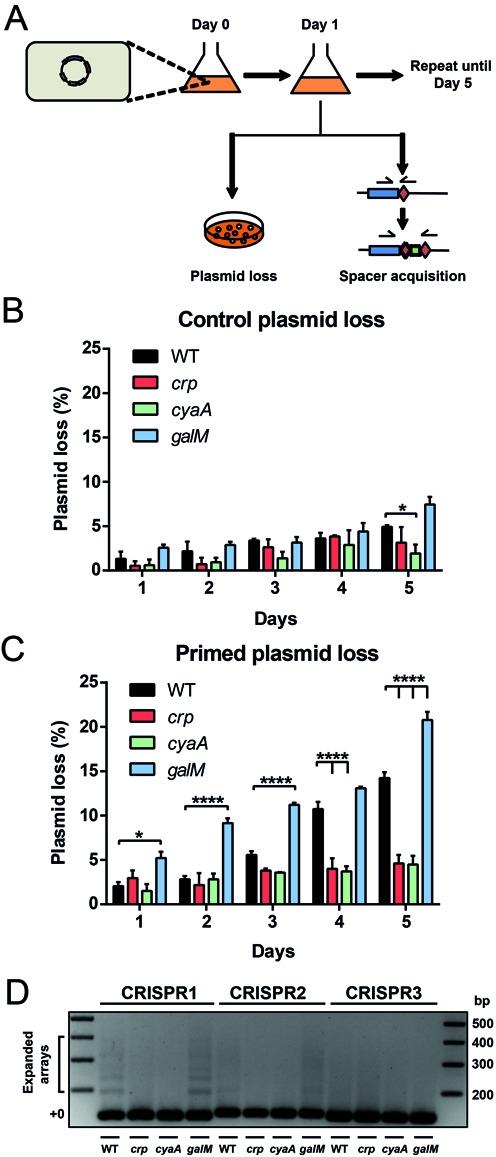
Altered *cas* expression influences adaptation. (**A**) Schematic of the plasmid loss assay. Relevant strains containing either a control (pPF571) or primed (pPF574) plasmid possessing a protospacer with a non-consensus PAM were grown in LB without antibiotic selection over 5 d (see Materials and Methods). Plasmid loss was scored visually using mCherry on LBA plates with 1 mM IPTG. Pooled genomic DNA at day 5 was screened for spacer acquisition in all CRISPRs. Plasmid loss of the (**B**) control plasmid (pPF571) or (**C**) primed plasmid (pPF574) from the WT (REM200), *crp* mutant (PCF112), *cyaA* mutant (PCF113) or *galM* mutant (PCF118) over 5 d. Data shown are the mean ± SD (*n* = 3). Statistical significance was calculated using Dunnett's multiple comparisons test (*P ≤ 0.05; ****P ≤ 0.0001). (**D**) PCRs to detect expansion of CRISPR arrays (CRISPR1–3) in the WT, *crp, cyaA* or *galM* mutants at day 5 (see Materials and Methods). Unexpanded CRISPR arrays correspond to the ‘+0’ label.

We have demonstrated that cAMP-CRP and GalM significantly influence the expression of the *cas* operon. However, a critical question is whether these transcriptional changes affect CRISPR-Cas adaptation and interference. To test the impact of cAMP-CRP and GalM on adaptation, cells were transformed with either a non-targeted control plasmid or a primed plasmid possessing a protospacer (with a mutated non-consensus PAM) corresponding to spacer 1 in CRISPR1 and passaged through successive days. The non-consensus PAM was previously shown to abolish initial interference, yet promoted primed acquisition of additional spacers into all three the chromosomal CRISPR arrays (17,28). Plasmid loss was assessed by scoring pink and white colonies using the plasmid-encoded mCherry (Figure 6A). Spacer acquisition was assessed via PCR of all CRISPRs from the entire population (Figure 6A). All strains containing the non-primed plasmid exhibited a minimal increase in plasmid loss up until day 5 with non-significant variation between all mutant strains and the WT control, except for *cyaA* which was reduced (Figure 6B). In contrast, the primed plasmid was progressively lost from WT cells to ∼14% by day 5 (Figure 6C), which was accompanied by detectable expansion of the CRISPR1 and CRISPR2 arrays (Figure 6D). Expansion of CRISPR3 was not detected, but this was expected as our previous work demonstrated that acquisition within this array is rare (∼3%) (17). Primed plasmid loss and spacer acquisition was not detectable in the *crp* or *cyaA* mutants, with levels remaining comparable to that of the non-primed plasmid (Figure 6C and D). Presumably, the reduced *cas* operon expression in the *crp* and *cyaA* mutants is insufficient to support priming. Interestingly, the *galM* mutant exhibited increased plasmid loss compared with the WT, reaching approximately ∼20% by day 5 (Figure 6C). As expected, plasmid loss was accompanied by CRISPR expansion (Figure 6D). Therefore, even when active in the WT, the CRISPR-Cas system does not function at a maximal, or saturated, level, and can be further stimulated. Taken together, these results demonstrate that CRP-cAMP is required for primed spacer acquisition by the Type I-F CRISPR-Cas system and that loss of GalM increases adaptation efficiency.

### Altered *cas* expression influences interference

**Figure 7.**
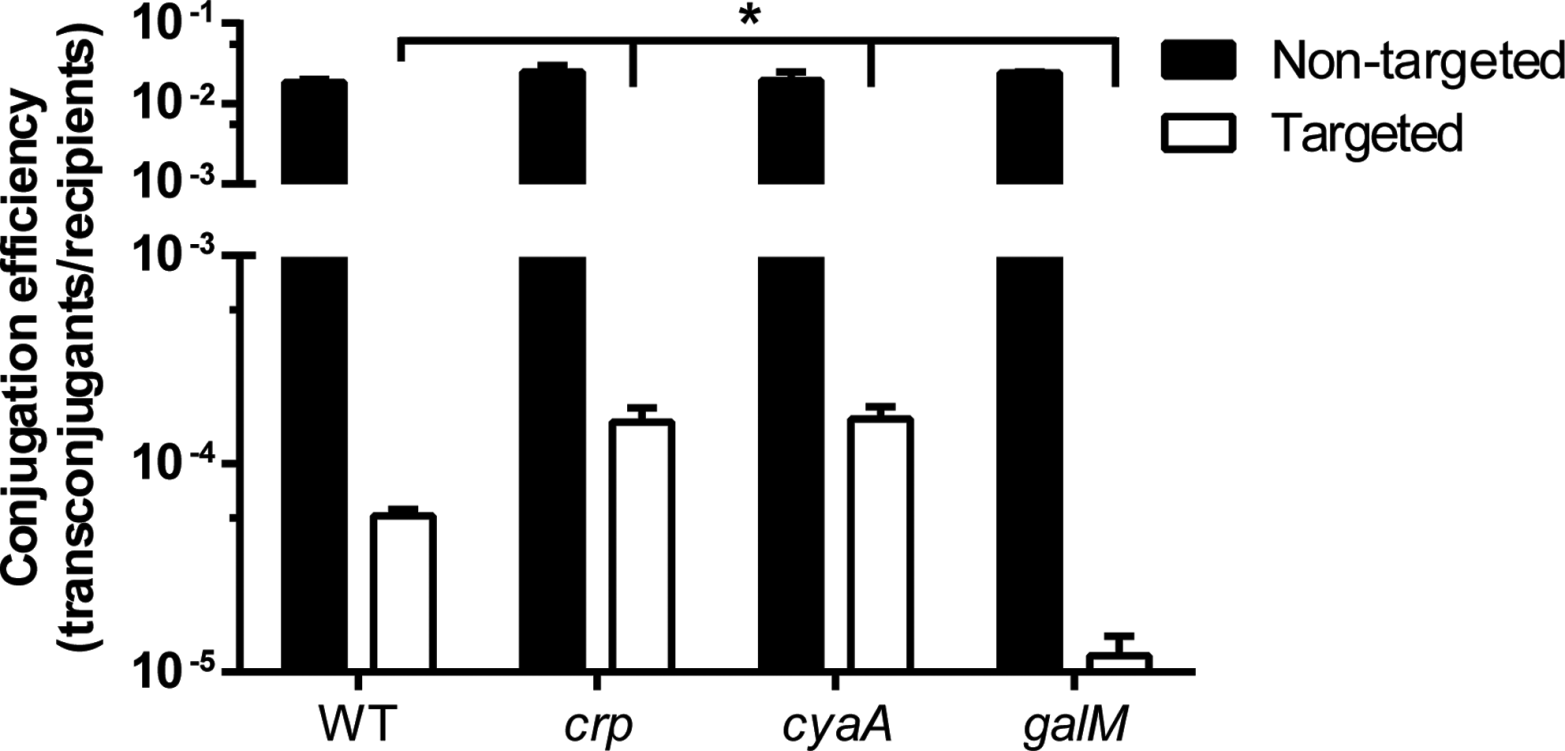
Altered *cas* expression influences interference. Conjugation efficiency of either non-targeted (pPF571) or CRISPR-Cas-targeted (pPF572) plasmids into the WT (REM200), *crp* mutant (PCF112), *cyaA* mutant (PCF113) or *galM* mutant (PCF118) (see Materials and Methods). Conjugation efficiency was scored as transconjugants/recipients. Data shown are the mean ± SD (*n* = 3). Statistical significance was calculated using Bonferroni's multiple comparisons test (*P ≤ 0.05).

Next, we wanted to test whether mutation of *crp, cyaA* and *galM* affected CRISPR-Cas interference. Conjugation assays were performed using an untargeted control plasmid and a CRISPR-Cas-targeted plasmid that contained a protospacer with a consensus GG PAM that is recognised by spacer 1 in CRISPR1 (17). Conjugation for the untargeted control plasmid was similar for all strains, indicating each had equivalent efficiency for conjugation (Figure 7). In the WT, CRISPR-Cas interfered with conjugation by ∼300-fold (Figure 7) compared with the untargeted plasmid. Conversely, in the *crp* and *cyaA* mutants, interference was >2-fold less severe, with only a ∼150-fold decrease in conjugation efficiency (Figure 7). The *galM* mutant exhibited a 7-fold increase in interference when compared with the WT, indicating that interference can be enhanced (Figure 7). Therefore, the degree of Type I-F CRISPR-Cas interference is influenced by *cas* operon expression.

## DISCUSSION

In *E. coli*, CRP is a global transcriptional regulator, responsible for controlling diverse processes from carbohydrate metabolism through to flagella synthesis (31,40,45–48). Our results establish a clear role for a pathway involving glucose, GalM and CRP-cAMP in regulating the *P. atrosepticum* Type I-F CRISPR-Cas system. These findings provide the first information about regulation of Type I-F systems. Control of CRISPR-Cas systems by CRP is not without precedent. *T. thermophilus* contains 12 CRISPR arrays belonging to Type I-E, III-A and III-B systems and CRP activates the Type I-E and Type III-A systems, (21,49). In contrast, the Type III-B system was repressed during phage infection, but independently of CRP. The *T. thermophilus* CRP binding site differs significantly from *E. coli* and *P. atrosepticum*. However, in common with the *P. atrosepticum* Type I-F system, the *T. thermophilus* CRP-box is centred between −38 and −45 and overlaps the −35 element, characteristic of Class II-dependent control (49). CRISPR-Cas activation by CRP in *T. thermophilus* specifically influences *cas* expression and not the CRISPRs (21), consistent with our results demonstrating that CRISPR1–3 promoter expression was unaltered in *crp* or *cyaA* mutants (Supplementary Figure S3).

An opposite role for CRP in regulation of the *E. coli* Type I-E system was suggested by Yang *et al*. (2014) (22). In *E. coli*, the CRP-box is located between −281 and −259 bp upstream of the *cse1* transcriptional start site and overlaps the binding site of the LeuO activator. It was proposed that cAMP increases CRP binding, which serves to limit activation by LeuO (i.e. CRP is an ‘anti-activator’). In contrast, the predicted *P. atrosepticum* Type I-F CRP-box is positioned optimally (−41.5) adjacent to the −35 site for Class II activation and is predicted to enhance RNAP binding and transcription. This emphasises a paradox regarding carbon source availability and its influence on the regulation of different CRISPR-Cas systems. Specifically, a glucose shortage causes *cas* activation in *P. atrosepticum*, while in *E. coli* elevated glucose limits CRP-cAMP competition for LeuO binding, promoting *cas* expression. This begs the question: why would such polar stimuli (glucose abundance vs. exhaustion) be involved in the expression of different CRISPR-Cas subtypes, especially as both species are gamma-proteobacteria and members of the Enterobacteriaceae? One explanation could be that the niches of these species are different. *E. coli* is present in animal gastrointestinal tracts, whereas *P. atrosepticum* thrives on plant surfaces, inside host stem and tuber tissues during maceration and within the surrounding rhizosphere (50). Plant cell wall degrading enzymes, in addition to horizontally acquired elements, such as the galactonate and gluconate metabolism islands within *P. atrosepticum*, highlight that it is capable of metabolising a diverse range of sugars (51). Differences in nutrient abundance and availability for *E. coli* and *P. atrosepticum* might account for why the same stimulus differentially regulates the expression of their CRISPR-Cas systems and indicates that control is niche-specific.

Given the *cas* response to glucose in our study, we propose that Type I-F expression is regulated by metabolic conditions within the cell, and that perturbation of normal metabolic flux influences defence against horizontally acquired genetic elements via CRP-cAMP. That GalM reduces *cas1* expression in a CRP-cAMP-dependent manner supports this hypothesis, as galactose metabolism ultimately results in the formation of glucose, specifically glucose-6-phosphate, limiting adenylate cyclase activation through the phosphotransferase system (42,43,52). Considering that only the α-D-galactose epimer of D-galactose enters into the Leloir galactose catabolic pathway, and approximately 36% of molecules exist in such a form in solution, the role of the GalM galactose mutarotase is essential for efficient metabolite processing (40,53,54).

Although there is a clear link between *cas* operon expression and catabolite repression, it is unlikely to be the only trigger of this adaptive response. A more plausible scenario involves an integrated and overlapping network which responds to multiple stresses to provide a robust means to detect invasion and elicit an appropriate response. Indeed, the BaeSR two-component system upregulates the *E. coli cas* operon in response to membrane stress (55). Another two-component system, VicRK, represses the Type II-A and activates the Type I-C systems in *Streptococcus mutans* in response to oxidative stress (56). In addition, the alarmone (p)ppGpp has been proposed as a possible signal through which LeuO activates the Type I-E system in *E. coli* in response to amino acid starvation (24). Given the diverse environmental conditions that *P. atrosepticum* is exposed to, it is tempting to speculate that various other regulators control CRISPR-Cas activity in addition to CRP-cAMP. By using 5′ RACE and RT-PCR, we previously showed that the *cas* and *csy* genes were expressed as a polycistronic mRNA from the *cas1* promoter (27). In this study we have discovered CRP-cAMP-dependent regulation at the *cas1* promoter. However, the *cas1* promoter might be controlled by other regulators and it is possible that an additional promoter exists within the 365 bp intergenic region between *cas2–3* and *csy1*, in which other regulators exert their control.

**Figure 8.**
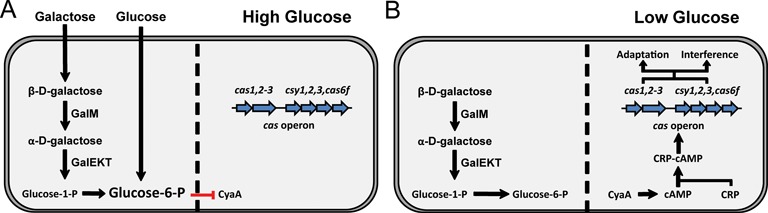
Model of Type I-F CRISPR-Cas regulation within *P. atrosepticum*. (**A**) During glucose abundance, adenylate cyclase (CyaA) activity is limited via the glucose phosphotransferase system. The reduced cAMP prevents activation of the *cas* operon via the CRP-cAMP complex. Intracellular glucose levels are bolstered during galactose metabolism, which requires GalM for the conversion of β-D-galactose into α-D-galactose. (**B**) Reduced glucose leads to activation of CyaA which facilitates cAMP production. In the presence of cAMP as a co-factor, dimeric CRP binds the *cas* promoter and activates the *cas* operon. The Cas1:Cas2–3 complex promotes the acquisition of new spacers into the CRISPRs while Csy1, Csy2, Csy3 and Cas6f form a complex with crRNA to recognise horizontally acquired elements, leading to the recruitment of Cas2–3 and interference.

We propose that during glucose abundance, expression of the Type I-F CRISPR-Cas system is restricted via repression of CyaA activity, preventing unnecessary expenditure of resources via *cas* operon expression (Figure 8A). Such control is reminiscent of catabolite repression seen during diauxic growth in *E. coli*, in which the presence of glucose limits *lac* operon expression in a cAMP-dependent manner (41,57,58). Regulation of CRISPR-Cas activity through an equivalent mechanism is likely to result in metabolic efficiency in the absence of invading elements. Indeed, there is considerable evidence that the acquisition and replication of extrachromosomal elements, such as phages and plasmids, can disrupt stable metabolic conditions (59–62). We predict that resulting nutrient deprivation signals are sensed by the *cas* operon through cAMP production, which promotes Csy complex formation to elicit a targeted response against the invader (Figure 8B).

Not all horizontally acquired elements are detrimental. For example, some confer a fitness benefit in a certain environment, such as those encoding antibiotic resistance in the presence of antibiotic exposure. With such a strong selection, cells with active CRISPR-Cas systems will be killed by the antibiotic and those able to maintain the element by CRISPR-Cas inactivation would survive (63). We propose that regulation might also play a role, in limiting the removal of beneficial mobile elements. A metabolic advantage provided by the element under certain conditions, such as antibiotic resistance in the presence of sub-inhibitory antibiotic concentrations, would serve to reduce CRISPR-Cas expression and minimise plasmid loss. Bacteriophages are obligate intracellular parasites that depend on bacterial resources for replication. Although many other factors are also involved, phage λ requires both CRP and cAMP for the induction or ‘switch’ from a prophage state in the chromosome to lytic replication, which ultimately destroys the bacterium (60,61,64–66). Indeed, if CRP-cAMP is involved in lysogenic phage induction, it is fitting that the same stimuli activates the CRISPR-Cas defence against these genetic elements. Since this might be a strategy used by other phages, it is not surprising that CRP-cAMP regulates CRISPR-Cas activity in multiple bacteria.

The changes in *cas* expression by mutation of *crp, cyaA* or *galM* correlated with changes in interference of conjugation, which supports experiments in *E. coli* using *hns* knockout strains or *leuO* overproduction constructs (24,67). Increases in LeuO and decreases in H-NS resulted in reduced sensitivity to phage infection with pre-existing anti-phage spacers, indicating that alteration of *cas* transcription results in changes in interference. Similarly, the influence of transcriptional regulators such as H-NS and Csa3a has been shown to influence adaptation in Type I-E and I-A CRISPR-Cas systems, respectively (12,16,68). In this study, we demonstrate that in the absence of CRP-cAMP spacer acquisition into CRISPR arrays of the Type I-F CRISPR-Cas system is undetectable, which has not been reported previously. Furthermore, elevated expression of the *cas* operon through *galM* mutation correlates with an increase in primed plasmid loss. This increase is likely via the de-repression of adenylate cyclase activity, resulting in an increase in *cas* operon components responsible for adaptation, specifically Cas1 and the Cas2–3 hybrid protein, which form an adaptation complex (69). The fact that plasmid loss was enhanced in the *galM* strain indicates that CRISPR-Cas activity is not maximal and that under permissive conditions (i.e. increased cAMP production), the system functions more efficiently to combat invaders.

In summary, expression of the *P. atrosepticum* Type I-F CRISPR-Cas system is tightly regulated in response to glucose by CRP-cAMP which activates the *cas1* promoter that drives expression of the entire *cas* operon. Upregulation of *cas* expression within this subtype correlates with increases in both interference and adaptation and thus facilitates an efficient defence response against horizontally acquired genetic elements.

## SUPPLEMENTARY DATA

Supplementary Data are available at NAR Online.

SUPPLEMENTARY DATA
