# Supplementary material for: Regulation of the Type I-F CRISPR-Cas system by CRP-cAMP and GalM controls spacer acquisition and interference
Source: Nucleic Acids Res. 2015 May 24;43(12):6038–48. doi: 10.1093/nar/gkv517 (PMC4499141; doi:10.1093/nar/gkv517)
Supplement: SUPPLEMENTARY DATA [file supp_43_12_6038__index.html]

Regulation of the Type I-F CRISPR-Cas system by CRP-cAMP and GalM controls spacer acquisition and interference — Regulation of the Type I-F CRISPR-Cas system by CRP-cAMP and GalM controls spacer acquisition and interference — SUPPLEMENTARY DATA 

# Regulation of the Type I-F CRISPR-Cas system by CRP-cAMP and GalM controls spacer acquisition and interference

## SUPPLEMENTARY DATA

- SUPPLEMENTARY DATA
