# Supplementary material for: Regulation of the Type I-F CRISPR-Cas system by CRP-cAMP and GalM controls spacer acquisition and interference
Source: Nucleic Acids Res. 2015 May 24;43(12):6038–48. doi: 10.1093/nar/gkv517 (PMC4499141; doi:10.1093/nar/gkv517)
Supplement: SUPPLEMENTARY DATA [file supp_gkv517_nar-00889-h-2015-File010.docx]

**Supplementary Information**

**Regulation of the Type I-F CRISPR-Cas system by CRP-cAMP and GalM controls spacer acquisition and interference**

Adrian G. Patterson, James T. Chang, Corinda Taylor and Peter C. Fineran*

Department of Microbiology and Immunology, University of Otago, PO Box 56, Dunedin 9054, New Zealand.

*For correspondence. E-mail. [peter.fineran@otago.ac.nz](mailto:peter.fineran@otago.ac.nz); Tel. +64 (0)3 479 7735; Fax. +64 (0)3 479 8540.

**SUPPLEMENTARY MATERIALS AND METHODS**

**Construction of *crp* and *cyaA* mutants via allelic exchange mutagenesis**

Deletion mutants of *crp* and *cyaA* in a *P. atrosepticum* REM200 (*lacZ*-) background were generated via allelic exchange mutagenesis (1, 2). Briefly, ~500 bp upstream and downstream regions were amplified using primers PF1282 + PF1285 and PF1290 + PF1293 for *crp* and PF1390 + PF1391 and PF1392 + PF1393 for *cyaA*. External primer pairs were used to generate an overlap of the *crp* flanks which was then cloned into the multi-cloning site (MCS) of pBlueScript II KS+, generating pPF602. The flanks of *cyaA* were fused to either end of a Cm resistance cassette amplified with PF432 and PF433 to facilitate positive selection of successful mutants and cloned into the MCS of pBlueScript II KS+, generating pPF623. Sequences were checked by PCR and sequencing (using PF217 and PF218) before sub-cloning into the MCS of pKNG101 using BamHI and XbaI, generating pPF604 and pPF624 for *crp* and *cyaA*, respectively. Plasmids were introduced into the *E. coli* CC118 λ*pir* via transformation and sequenced using PF213 and PF214.

Deletion constructs were conjugated into *P. atrosepticum* REM200 via tri-parental mating using the *E. coli* CC118 λ*pir* pPF604 or pPF624 strains as donors and *E. coli* HH26 (pNJ5000) as a helper. Briefly, 10 µl of washed recipient, donor and helper overnight cultures were combined and spotted on LBA and incubated at 25°C for 24 h. The mating spot was then streaked on minimal medium agar plates (containing 0.2% glucose (w v^-1^) and Sm for *crp* or Sm + Cm for *cyaA*) and incubated at 25°C for 4-5 d. Colonies were then grown overnight in LB, diluted 1:100 with 1x phosphate buffered saline (PBS) and 10 μl were plated onto minimal medium containing 10% (w v^-1^) sucrose (with Cm for *cyaA*). Plates were incubated at 25°C for 4-5 d and colonies checked for sensitivity to Sm to verify loss of the pKNG101 plasmid. The absence of pNJ5000 was determined by checking for Tc sensitivity. Mutants were confirmed by PCR and sequencing. The *crp* mutant was constructed without an antibiotic resistance marker to enable the generation of compatible double and triple mutants. The same rationale was used to select the Cm^R^ marker in *cyaA*.

**Generalised transduction of marked mutations**

The *crp cyaA*, *crp galM* and *cyaA galM* double mutants and the *crp cyaA galM* triple mutant were generated by generalised transduction using ΦTE (3). High titre ΦTE phage lysates were prepared using donor bacteria possessing marked *cyaA* (Cm^R^) and *galM* (Km^R^) mutations and used to infect ~10^10^ cells of recipient strains at a multiplicity of infection of 0.1. Cells were incubated for 45 min in 10 ml of LB at 90 rpm before washing 3× with fresh LB to remove excess phage. Bacteria were plated on LBA with Cm, Km or Cm + Km and incubated for 24 h to select for transductants. The resulting transductants were confirmed by PCR and sequencing.

**CRISPR*-* and *cas-lacZ* reporter construction**

Promoters for *cas1*, CRISPR1, CRISPR2 and CRISPR3 were amplified by PCR from *P. atrosepticum* using primers PF1308 + PF1309, PF1314 + PF1315, PF1316 + PF1317 and PF1318 + PF1319, respectively. The products were cloned into pVIK107 (4) after digestion with EcoRI and SalI (*cas1*, CRISPR1 and CRISPR3) or MfeI and SalI (CRISPR2). A *cas1*-ΔCRP-box reporter was prepared, in which the first CRP half-site was altered with a SmaI site, while the second half-site was altered to contain a HindIII site. This was achieved using the external primers PF1309 and PF1373 in addition to PF1374 and PF1375 which bind at the -35 region and contain the CRP-box mutations. The resulting pVIK107 plasmids were digested with NcoI and BglII restriction enzymes to remove the Km resistance cassette. The Tc resistance cassette was then amplified using PF1364 and PF1372 from pTRB31 and ligated into the NcoI and BglII-digested pVIK107-promoter vectors. *E. coli* CC118 λ*pir* was transformed with the ligations and plated on LBA with Tc. Plasmids were sequenced to verify the promoters and resistance cassette. *E. coli* S-17 λ*pir* was used to conjugate the plasmids into REM200 and mutant derivatives via bi-parental mating. Briefly, 15 µl of washed recipient and donor overnight cultures were combined and spotted on LBA and incubated at 25°C for 24 h. Spots were streaked on LBA with Tc in addition to antibiotics for specific mutant strains, or 0.2% (w v^-1^) minimal glucose medium with Tc for the unmarked isolates. The strains were confirmed by PCR and sequencing.

**Generation of CRP, CyaA and GalM complementation constructs**

PCR was used to amplify the *crp*, *cyaA* and *galM* genes using primers (PF1220 + PF1221 for *crp*, PF1280 + PF1281 for *cyaA* and PF1214 + PF1215 for *galM*) containing an optimal ribosome binding site preceding the start codon. Products then underwent restriction digest (EcoRI and XmaI for *crp*, EcoRI and SmaI for *cyaA* and SmaI and PstI for *galM*) and were ligated into the MCS of pQE-80L for *crp* and *cyaA* or into pQE-80L-oriT for *galM*. Ligations were transformed into *E. coli* S-17 λ*pir* via heat shock and plated on LBA with Ap. Plasmids were confirmed by sequencing with PF209 and PF210. Plasmids were induced with 0.1 mM IPTG during complementation analysis.

**Supplementary Table S1**. Bacterial strains used in this study.

| **Strain** | **Genotype/Phenotype** | **Reference** |
| --- | --- | --- |
| ***Escherichia coli*** | |  |
| BW20767 | RP4-2-*tet*:Mu-1*kan*::Tn7 integrant *leu*-*63*::IS*10* *recA1* *creC510* *hsdR17* *endA1* *zbf-5* *uidA*(ΔMluI):*pir*^+^ *thi* | (5) |
| CC118 λ*pir* | *araD*, Δ(*ara*, *leu*), Δ*lacZ*74, *phoA*20, *galK*, *thi-1*, *rspE*, *rpoB*, *argE*, *recA*1, λpir | (6) |
| DH5α | F^-^, φ80Δd*lacZ*M15, Δ(*lacZYA*–*argF*)U169, *endA1*, *recA1*, *hsd*R17 (r_K_^-^m_K_^+^), *deoR*, *thi-1*, *sup*E44, λ^-^, *gyr*A96, *rel*A1 | Gibco/BRL |
| HH26 | Marker exchange host for mobilization of suicide vector by plasmid pNJ5000 | (7) |
| S17-1 λ*pir* | *recA, pro,hsdR, recA::*RP4-2-Tc::Mu, λ*pir,* Tmp^R^, Sp^R^, Sm^R^ | (8) |
| ***Pectobacterium atrosepticum*** | | |
| REM200 | *lacZ*- derivative of *Pectobacterium atrosepticum* SCRI1043. Referred to as wildtype in this study | Rita Monson, unpublished |
| ***REM200 derivatives*** | | |
| PCF79 | Δ*cas*::*lacZcat*, used for transposon mutagenesis, Cm^R^ | (9) |
| PCF85 | Δ*cas*::*lacZcat, galM*::Tn-DS1028*uidA*Km, Cm^R^, Km^R^ | This study |
| PCF112 | Δ*crp* | This study |
| PCF113 | Δ*cyaA*::*cat*, Cm^R^ | This study |
| PCF116 | Δ*crp,* Δ*cyaA*::*cat*, Cm^R^ | This study |
| PCF117 | Δ*crp,* *galM*::Tn-DS1028*uidA*Km, Km^R^ | This study |
| PCF118 | *galM*::Tn-DS1028*uidA*Km, Km^R^ | This study |
| PCF119 | Δ*crp,* Δ*cyaA*::*cat*, *galM*::Tn-DS1028*uidA*Km, Cm^R^, Km^R^ | This study |
| PCF120 | Δ*cyaA*::*cat,* *galM*::Tn-DS1028*uidA*Km, Cm^R^, Km^R^ | This study |
| PCF123 | *cas1*_pro_::pPF705 (*cas-lacZ*), Tc^R^ | This study |
| PCF124 | Δ*crp,* *cas1*_pro_:: pPF705 (*cas-lacZ*), Tc^R^ | This study |
| PCF125 | Δ*cyaA*::*cat,* *cas1*_pro_:: pPF705 (*cas-lacZ*), Cm^R^, Tc^R^ | This study |
| PCF126 | *galM*::Tn-DS1028*uidA*Km, *cas1*_pro_:: pPF705 (*cas-lacZ*), Km^R^, Tc^R^ | This study |
| PCF127 | Δ*crp,* Δ*cyaA*::*cat,* *cas1*_pro_:: pPF705 (*cas-lacZ*), Cm^R^, Tc^R^ | This study |
| PCF128 | Δ*crp,* *galM*::Tn-DS1028*uidA*Km, *cas1*_pro_:: pPF705 (*cas-lacZ*), Km^R^, Tc^R^ | This study |
| PCF129 | Δ*cyaA*::*cat,* *galM*::Tn-DS1028*uidA*Km, *cas1*_pro_:: pPF705 (*cas-lacZ*), Cm^R^, Km^R^, Tc^R^ | This study |
| PCF130 | Δ*crp,* Δ*cyaA*::*cat,* *galM*::Tn-DS1028*uidA*Km, *cas1*_pro_:: pPF705 (*cas-lacZ*), Cm^R^, Km^R^, Tc^R^ | This study |
| PCF133 | *cas1*_pro_:: pPF706 (*cas-*ΔBS*-lacZ*), Tc^R^ | This study |
| PCF134 | Δ*crp,* *cas1*_pro_:: pPF706 (*cas-*ΔBS*-lacZ*), *cas1 pro+*, Tc^R^ | This study |
| PCF135 | Δ*cyaA*::*cat,* *cas1*_pro_:: pPF706 (*cas-*ΔBS*-lacZ*), Cm^R^, Tc^R^ | This study |
| PCF136 | *galM*::Tn-DS1028*uidA*Km, *cas1*_pro_:: pPF706 (*cas-*ΔBS*-lacZ*), Km^R^, Tc^R^ | This study |
| PCF137 | Δ*crp,* Δ*cyaA*::*cat,* *cas1*_pro_:: pPF706 (*cas-*ΔBS*-lacZ*), Cm^R^, Tc^R^ | This study |
| PCF138 | Δ*crp,* *galM*::Tn-DS1028*uidA*Km, *cas1*_pro_:: pPF706 (*cas-*ΔBS*-lacZ*), Km^R^, Tc^R^ | This study |
| PCF139 | Δ*cyaA*::*cat,* *galM*::Tn-DS1028*uidA*Km, *cas1*_pro_:: pPF706 (*cas-*ΔBS*-lacZ*), Cm^R^, Km^R^, Tc^R^ | This study |
| PCF140 | Δ*crp,* Δ*cyaA*::*cat,* *galM*::Tn-DS1028*uidA*Km, *cas1*_pro_:: pPF706 (*cas-*ΔBS*-lacZ*), Cm^R^, Km^R^, Tc^R^ | This study |
| PCF143 | CRISPR1_pro_:: pPF707 (CRISPR1*-lacZ*), Tc^R^ | This study |
| PCF144 | Δ*crp,* CRISPR1_pro_:: pPF707 (CRISPR1*-lacZ*), Tc^R^ | This study |
| PCF145 | Δ*cyaA*::*cat,* CRISPR1_pro_:: pPF707 (CRISPR1*-lacZ*), Cm^R^, Tc^R^ | This study |
| PCF146 | *galM*::Tn-DS1028*uidA*Km, CRISPR1_pro_:: pPF707 (CRISPR1*-lacZ*), Km^R^, Tc^R^ | This study |
| PCF147 | Δ*crp* Δ*cyaA*::*cat,* CRISPR1_pro_:: pPF707 (CRISPR1*-lacZ*), Cm^R^, Tc^R^ | This study |
| PCF148 | Δ*crp* *galM*::Tn-DS1028*uidA*Km, CRISPR1_pro_:: pPF707 (CRISPR1*-lacZ*), Km^R^, Tc^R^ | This study |
| PCF149 | Δ*cyaA*::*cat*, *galM*::Tn-DS1028*uidA*Km, CRISPR1_pro_:: pPF707 (CRISPR1*-lacZ*), Cm^R^, Km^R^, Tc^R^ | This study |
| PCF150 | Δ*crp*, Δ*cyaA*::*cat*, *galM*::Tn-DS1028*uidA*Km, CRISPR1_pro_:: pPF707 (CRISPR1*-lacZ*), Cm^R^, Km^R^, Tc^R^ | This study |
| PCF153 | CRISPR2_pro_:: pPF708 (CRISPR2*-lacZ*) Tc^R^ | This study |
| PCF154 | Δ*crp,* CRISPR2_pro_:: pPF708 (CRISPR2*-lacZ*), Tc^R^ | This study |
| PCF155 | Δ*cyaA*::*cat,* CRISPR2_pro_:: pPF708 (CRISPR2*-lacZ*), Cm^R^, Tc^R^ | This study |
| PCF156 | *galM*::Tn-DS1028*uidA*Km. CRISPR2_pro_:: pPF708 (CRISPR2*-lacZ*), Km^R^, Tc^R^ | This study |
| PCF157 | Δ*crp*, Δ*cyaA*::*cat,* CRISPR2_pro_:: pPF708 (CRISPR2*-lacZ*), Cm^R^, Tc^R^ | This study |
| PCF158 | Δ*crp*, *galM*::Tn-DS1028*uidA*Km, CRISPR2_pro_:: pPF708 (CRISPR2*-lacZ*), Km^R^, Tc^R^ | This study |
| PCF159 | Δ*cyaA*::*cat*, *galM*::Tn-DS1028*uidA*Km, CRISPR2_pro_:: pPF708 (CRISPR2*-lacZ*), Cm^R^, Km^R^, Tc^R^ | This study |
| PCF160 | Δ*crp*, Δ*cyaA*::*cat*, *galM*::Tn-DS1028*uidA*Km, CRISPR2_pro_:: pPF708 (CRISPR2*-lacZ*), Cm^R^, Km^R^, Tc^R^ | This study |
| PCF163 | CRISPR3_pro_:: pPF709 (CRISPR3*-lacZ*), Tc^R^ | This study |
| PCF164 | Δ*crp,* CRISPR3_pro_:: pPF709 (CRISPR3*-lacZ*), Tc^R^ | This study |
| PCF165 | Δ*cyaA*::*cat*, CRISPR3_pro_:: pPF709 (CRISPR3*-lacZ*), Cm^R^, Tc^R^ | This study |
| PCF166 | *galM*::Tn-DS1028*uidA*Km, CRISPR3_pro_:: pPF709 (CRISPR3*-lacZ*), Km^R^, Tc^R^ | This study |
| PCF167 | Δ*crp*, Δ*cyaA*::*cat*, CRISPR3_pro_:: pPF709 (CRISPR3*-lacZ*), Cm^R^, Tc^R^ | This study |
| PCF168 | Δ*crp*, *galM*::Tn-DS1028*uidA*Km. CRISPR3_pro_:: pPF709 (CRISPR3*-lacZ*) Km^R^, Tc^R^ | This study |
| PCF169 | Δ*cyaA*::*cat*, *galM*::Tn-DS1028*uidA*Km, CRISPR3_pro_:: pPF709 (CRISPR3*-lacZ*) Cm^R^, Km^R^, Tc^R^ | This study |
| PCF170 | Δ*crp*, Δ*cyaA*::*cat*, *galM*::Tn-DS1028*uidA*Km, CRISPR3_pro_:: pPF709 (CRISPR3*-lacZ*)Cm^R^, Km^R^, Tc^R^ | This study |
| PCF173 | Δ*cas*::*lacZcat, crp*_pro_::Tn-DS1028*uidA*Km, Cm^R^, Km^R^ | This study |

**Supplementary Table S2**. Plasmids used in this study.

| **Plasmid** | **Description** | **Reference** |
| --- | --- | --- |
| pBluescript II KS+ | Cloning vector, ColE1 replicon, Ap^R^ | Stratagene |
| pKNG101 | Suicide vector, *sacBR*, *mobRK2*, R6K ori, Sm^R^ | (1) |
| pKRCPN2 | Conjugative plasmid containing Tn-DS1028*uidA*Km (mini-Tn5-based), R6K ori, Km^R^, Tc^R^ | Kevin Roberts, unpublished |
| pNJ5000 | Mobilising plasmid for marker exchange, Tc^R^ | ([46](#_ENREF_46)) |
| pPF571 | Non-targeted control plasmid, pQE-80L-oriT-mCherry-derivative, Tc^R^ | (10) |
| pPF572 | CRISPR1 spacer 1 F interference plasmid, pQE-80L-oriT-mCherry-derivative, Tc^R^ | This study |
| pPF574 | CRISPR1 spacer 1 F priming plasmid, pQE-80L-oriT-mCherry-derivative, Tc^R^ | (10) |
| pPF600 | CRP expression vector, pQE-80L derivative, Ap^R^ | This study |
| pPF604 | Suicide vector for *crp* mutant, pKNG101-derivative, Sp^R^/Sm^R^ | This study |
| pPF616 | pVIK107 derivative with Km^R^ replaced by Tc^R^, Tc^R^ | This study |
| pPF620 | GalM expression vector, pQE-80L-oriT-derivative, Ap^R^ | This study |
| pPF622 | CyaA expression vector, pQE-80L-derivative, Ap^R^ | This study |
| pPF624 | Suicide vector for *cyaA* mutant, pKNG101 derivative, Cm^R^, Sp^R^/Sm^R^ | This study |
| pPF705 | *cas1-lacZ* expression vector*,* pVIK107-Tc^R^ with *cas1* promoter cloned into MCS. For integration into the *cas1* promoter region | This study |
| pPF706 | *cas1-*ΔBS*-lacZ* expression vector, pVIK107-Tc^R^ with *cas1* promoter with a mutated CRP binding consensus cloned into MCS. For integration into the *cas1* promoter region | This study |
| pPF707 | *CRISPR1-lacZ* expression vector, pVIK107-Tc^R^ with *CRISPR1* promoter cloned into MCS. For integration into the *CRISPR1* promoter region | This study |
| pPF708 | *CRISPR2-lacZ* expression vector, pVIK107-Tc^R^ with *CRISPR2* promoter cloned into MCS. For integration into the *CRISPR2* promoter region | This study |
| pPF709 | *CRISPR3-lacZ* expression vector, pVIK107-Tc^R^ with *CRISPR3* promoter cloned into MCS. For integration into the *CRISPR3* promoter region | This study |
| pQE-80L | QIAexpress pQE vectors, Ap^R^, ColE1 ori, MCS under T5 promoter control. Able to introduce His_6_ tag at either N or C terminal end. | QIAGEN |
| pQE-80L-oriT | pQE-80L derivative, *oriT*, Ap^R^ | (11) |
| pQE-80L-oriT-mCherry | pQE-80L derivative, mCherry, *oriT,* Ap^R^ | Josh Ramsay, unpublished |
| pTRB30 | pQE-80L derivative, Ap^R^ replaced by Km^R^, Km^R^ | Tim Blower, unpublished |
| pTRB31 | pQE-80L derivative, Ap^R^ replaced by Tc^R^, Tc^R^ | Tim Blower, unpublished |
| pVIK107 | Integrative vector for *cis* merodipoloid mutants. Allows for construction of reporter mutants in most genes, Km^R^ | (4) |

**Supplementary Table S3**. Oligonucleotide primers used in this study.

| **Name** | **Sequence (5'-3') (**Restriction site(s) underlined) | **Notes** |
| --- | --- | --- |
| PF209 | TCGTCTTCACCTCGAGAAATC | F^[[1]](#footnote-1)^ for pQE-80L MCS |
| PF210 | GTCATTACTGGATCTATCAACAGG | R^[[2]](#footnote-2)^ for pQE-80L MCS |
| PF213 | CAACTTAACGTAAAAACAACTTCAGA | F for pKNG101 MCS |
| PF214 | TACACTTCCGCTCAGGTCCTTGTCCT | R for pKNG101 MCS |
| PF217 | CGACGTAAAACGACGGCCAGT | F for pBluescript II KS+ MCS |
| PF218 | GGAAACAGCTATGACCATG | R for pBluescript II KS+ MCS |
| PF294 | CTTGCTCAATCAATCACCG | Nested primer for Km end of Tn *uidA*Km (Second PCR) |
| PF337 | GGTTAATTGGTTGTAACACTGGC | External primer for Km end of Tn *uidA*Km (First PCR) |
| PF338 | ATCTGCATCGGCGAACTGAT | Primer for random primed PCR from *uidA* |
| PF432 | TTTGTCGACATACCGGGAAGCCCTGGG | R for Cm cassette (SalI) |
| PF433 | TTTAAGCTTAGGCGTTTAAGGGCACCA | F for Cm cassette (HindIII) |
| PF1209 | GTCGAGTTTTTTGATTTCACG | Primer for random primed PCR from *uidA* |
| PF1214 | ATACCCGGGAGGAGGACAGGGATGTTGAATGAAAGTCTTGG | F for *galM* cloning (SmaI) |
| PF1215 | GATCTGCAGTTACTGGATGAGAAATTGG | R for *galM* cloning (PstI) |
| PF1220 | ATAGAATTCCAGAGGATGATAGCGAATGG | F for *crp* cloning (EcoRI) |
| PF1221 | GATCCCGGGTTAGCGAGTGCCGTAAACG | R for *crp* cloning (XmaI) |
| PF1280 | ATAGAATTCGGGGTTTTTCTAGGCACTG | F for *cyaA* cloning (EcoRI) |
| PF1281 | GATCCCGGGGATATTCTAAACATCGGCGAC | F for *cyaA* cloning (SmaI) |
| PF1282 | ATAGGATCCACATCAACACCATTTCCATC | F for Δ*crp* left flank (BamHI) |
| PF1285 | GTATCGCGACAGGGGTATTCACTGCGCAAATTACAG | R for Δ*crp* left flank |
| PF1290 | ACCCCTGTCGCGATAC | F for Δ*crp* right flank |
| PF1293 | GATTCTAGATACACGGCACCACCTATG | R for Δ*crp* right flank (XbaI) |
| PF1308 | AGAGAATTCTGTAGCCCGATCACTCTAAC | F for *cas1* promoter (EcoRI) |
| PF1309 | GATGTCGACCATAATGTATTTTCTTCCGTAATAG | R for *cas1* promoter (SalI) |
| PF1314 | AGAGAATTCAATGTATTTTCTTCCGTAATAG | F for CRISPR1 promoter (EcoRI) |
| PF1315 | GATGTCGACCATAGCTGTTTCCTTGTAGCCCGATCACTCTAAC | R CRISPR1 promoter (SalI) |
| PF1316 | AGACAATTGAATGCACCTCCTCTTAATACAG | F for CRISPR2 promoter (MfeI) |
| PF1317 | GATGTCGACCATAGCTGTTTCCTCGTTCCTATTTTACGGTAGTTACC | R CRISPR2 promoter (SalI) |
| PF1318 | AGAGAATTCGCCGTTTGTGCAAATAAAG | F for CRISPR3 promoter (EcoRI) |
| PF1319 | GATGTCGACCATAGCTGTTTCCTTGTTAAATTTTCACACTAACAACC | R CRISPR3 promoter (SalI) |
| PF1320 | CGACGCTCTGAGGTTAATTTC | F for *crp* screening |
| PF1321 | ACGCCGGATATCCTTACAAC | R for *crp* screening |
| PF1355 | CAAAGCAATTTTCAGTGACAC | F pVIK107 MCS primer |
| PF1356 | GGGCCTCTTCGCTATTAC | R pVIK107 MCS primer |
| PF1364 | TTTTCCATGG GTCTGACGCTCAGTGGAACG | R for resistance cassettes (NcoI) |
| PF1365 | TTTTCCATGGACCGCAGAGACTATCGATACGGTCTGGACGGATGC GTCTGACGCTCAGTGGAACG | F containing protospacer in forward orientation for pPF572 |
| PF1372 | ATAAGATCTTGTTATCCGCTCACAAAGC | F for cloning Resistance cassettes (BglII) |
| PF1373 | AGAGAATTCTTGCTCGTCTGTCAAATTG | F for Δ*crp* pro (F PCR product) promoter fusion into pVIK107, around 500 bp away from *crp* binding site |
| PF1374 | AAGCTTATCCCGGGCCGGATTAAAAATCAATGAGTTACAG | R for Δ *crp* pro promoter fusion product into pVIK107, contains tail for overlap (SmaI, HindIII) |
| PF1375 | GGCCCGGGATAAGCTTAATAAAAAGGGTCTACGTTTGG | F for Δ *crp* pro (R PCR product) promoter fusion into pVIK107, contains tail for overlap (HindIII, SmaI) |
| PF1390 | GATTCTAGATGGCAATTCTAATAATATTGGC | F for Δ*cyaA* left flank (XbaI) |
| PF1390 | GATTCTAGATGGCAATTCTAATAATATTGGC | R for Δ*cyaA* left flank |
| PF1392 | CTTAAACGCCTAAGCTTAAA TCACTGCCAGACACTCG | F for Δ*cyaA* right flank |
| PF1393 | ATAGGATCCAAGCTGAGAAGCCTGACTAGC | R for Δ*cyaA* right flank (BamHI) |
| PF1403 | AGCTCCTGAAAATCTCGATAAC | Cm cassette outwards |
| PF1461 | ACAACCCGTTAGAGTGATCGGGCTAC | F for CRISPR1 leader |
| PF1464 | CAAGAGAACTACCGTAAAATAGGAACGG | F for CRISPR2 leader |
| PF1467 | TCGACGTGTTAGTGTGAAAATTTAACAGTTC | F for CRISPR3 leader |
| PF1470 | CTCAGG GGGATTCTACAACCCTAATTTC | R for CRISPR1 spacer 2 |
| PF1473 | TGGTCG CTTTAAGCGCATGTCGGTC | R for CRISPR2 spacer 2 |
| PF1476 | ACGCAT CAGAAAGCCGACTTCAATC | R for CRISPR3 spacer 2 |
| PF1540 | GCGCTAGCCGTAACAATC | F for *cyaA* sequencing |


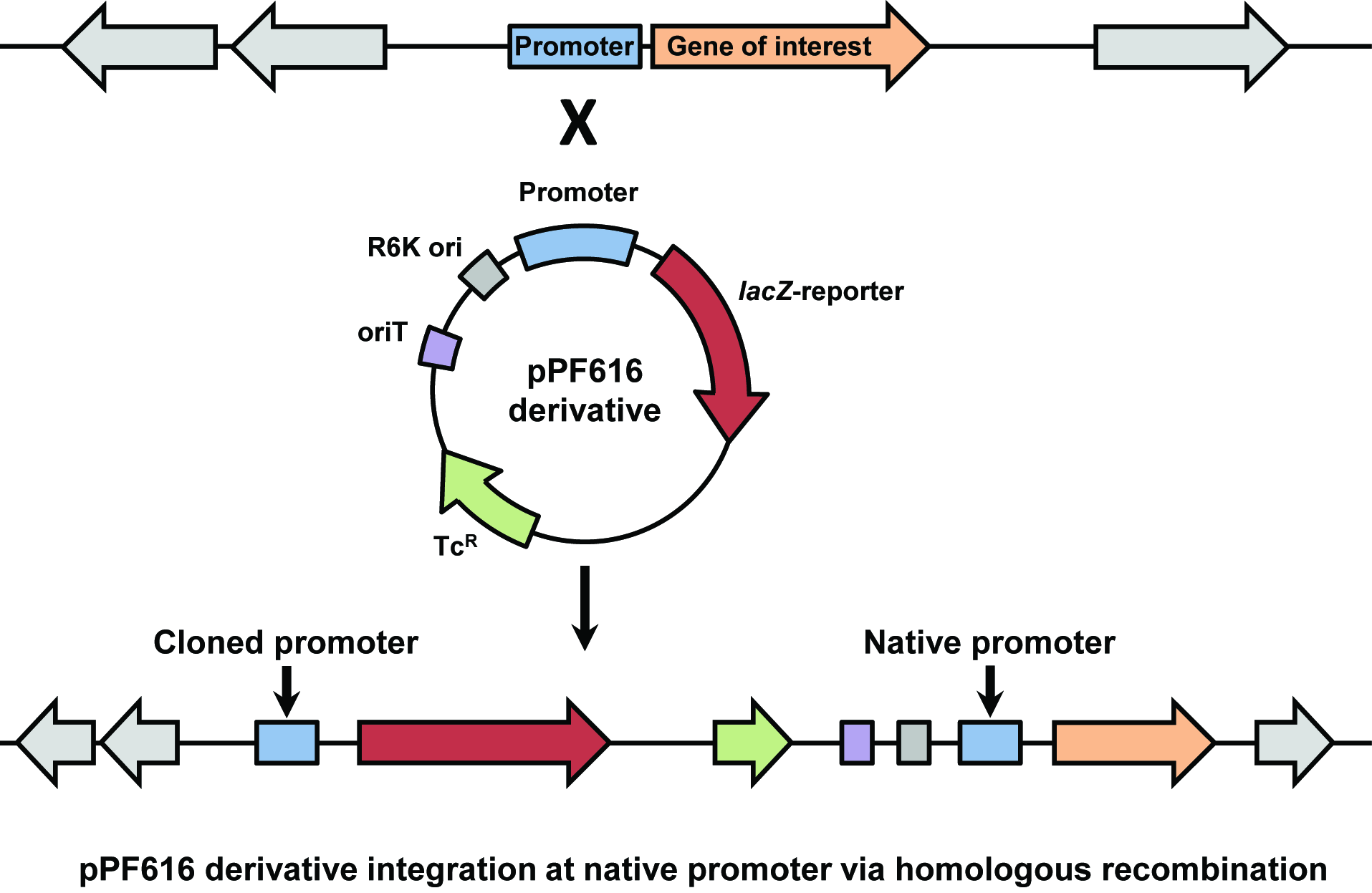


**Supplementary Figure 1.** Schematic of *lacZ*-reporter mutant construction. Promoters of interest were cloned into the multi-cloning site (MCS) of pPF616 to generate transcriptional *lacZ*-reporters. Replication of pPF616 independently of the bacterial chromosome requires the presence of the λ pir protein for activation of the R6K ori. In the absence λ pir, pPF616 may undergo homologous recombination at the cloned promoter region to generate a *cis*-merodiploid strain possessing the cloned promoter linked to the *lacZ*-reporter, in addition to the intact native promoter and downstream gene of interest. Maintanence of the integrated plasmid is achieved via antibiotic selection and analysis of β-galactosidase activity allows for quantification of promoter expression.


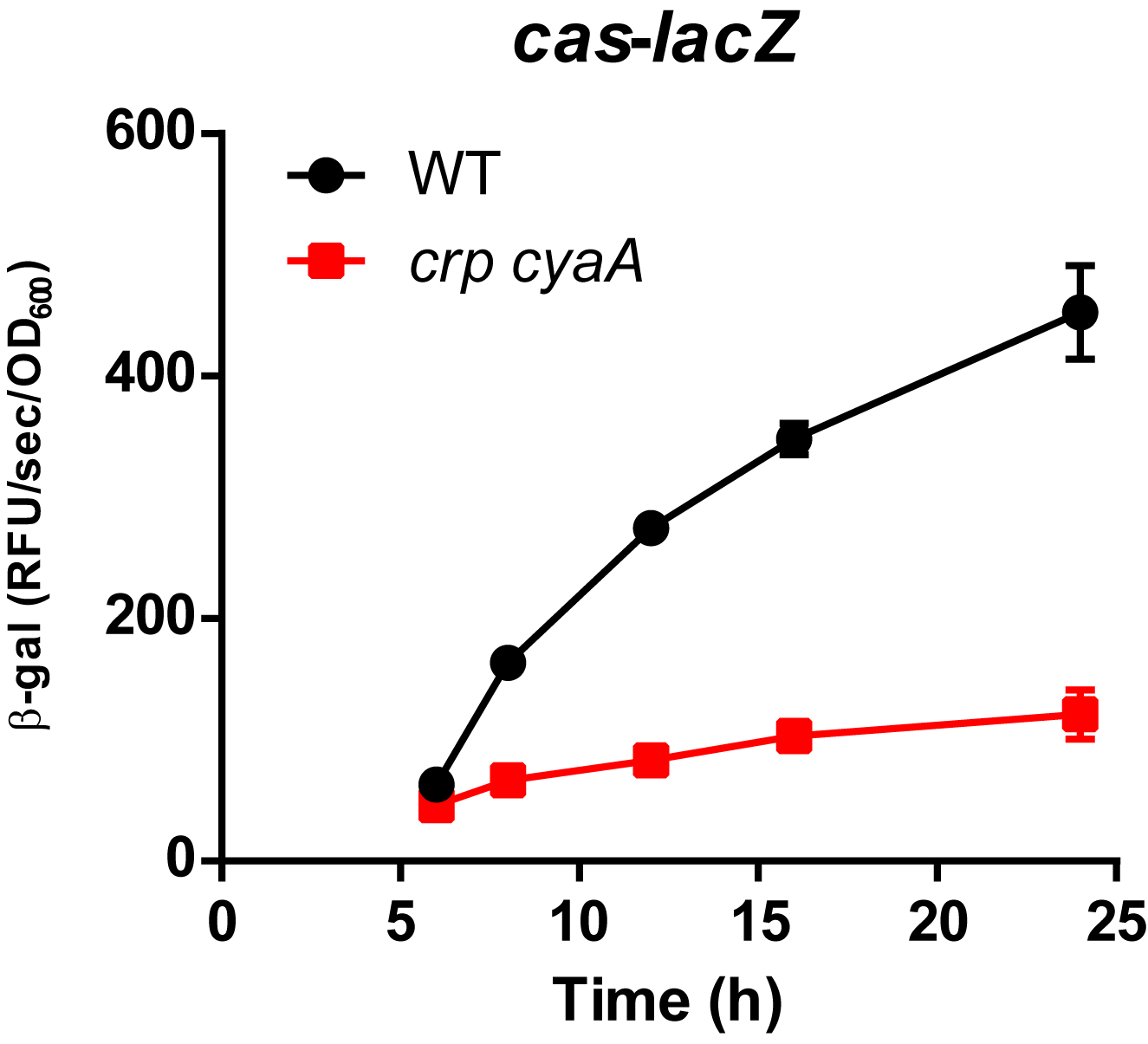


**Supplementary Figure 2.** Expression of *cas1* in the *crp cyaA* double mutant*.* Expression of the *cas1* promoter in the WT (PCF123, black), or *crp cyaA* mutant (PCF127, red) measured using an integrative *cas1*-*lacZ* reporter (pPF705). Data shown is the mean ± SD (*n* = 3).


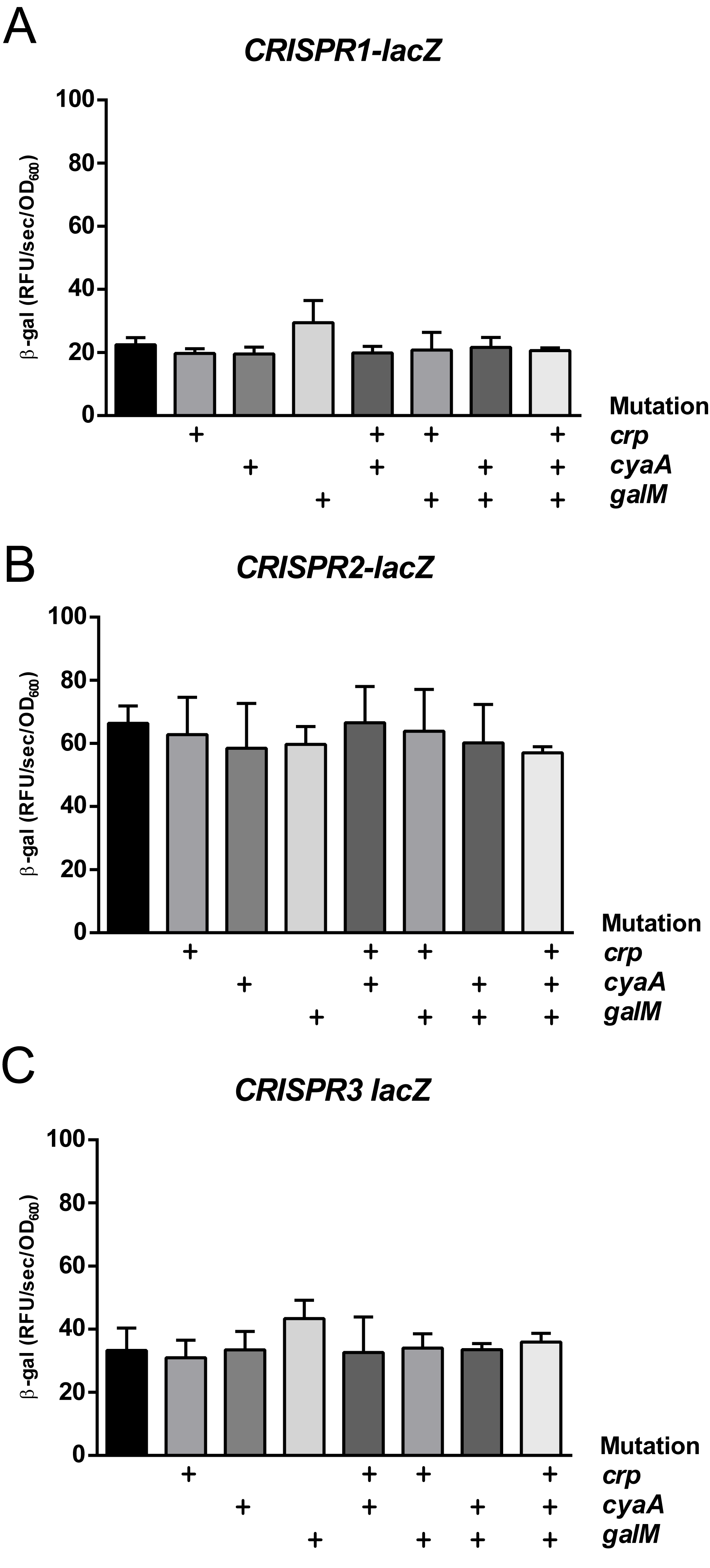


**Supplementary Figure 3.** CRISPR1, 2 and 3 array expression for various strains*.* Expression of the (A) CRISPR1, (B) CRISPR2 and (C) CRISPR3 array promoters in the WT (strains PCF143, PCF153, PCF163), *crp* (strains PCF144, PCF154, PCF164), *cyaA* (PCF145, PCF155, PCF165), *galM* (PCF146, PCF156, PCF166), *crp cyaA* (PCF147, PCF157, PCF167), *crp galM* (PCF148, PCF158, PCF168), *cyaA galM* (PCF149, PCF159, PCF169) or *crp cyaA galM* (PCF150, PCF160, PCF170) backgrounds at 24 h post inoculation. Expression of each array was measured using integrative CRISPR1, CRISPR2 or CRISPR3-*lacZ* reporters, respectively (pPF707, pPF708, pPG709). Data shown is the mean ± SD (*n* = 3).

**References**

1. Kaniga,K., Delor,I. and Cornelis,G.R. (1991) A wide-host-range suicide vector for improving reverse genetics in Gram-negative bacteria: inactivation of the *blaA* gene of *Yersinia enterocolitica*. *Gene*, **109**, 137–141.

2. Fineran,P.C., Everson,L., Slater,H. and Salmond,G.P.C. (2005) A GntR family transcriptional regulator (PigT) controls gluconate-mediated repression and defines a new, independent pathway for regulation of the tripyrrole antibiotic, prodigiosin, in *Serratia*. *Microbiology*, **151**, 3833–45.

3. Blower,T.R., Evans,T.J., Przybilski,R., Fineran,P.C. and Salmond,G.P.C. (2012) Viral evasion of a bacterial suicide system by RNA-based molecular mimicry enables infectious altruism. *PLoS Genet.*, **8**, e1003023.

4. Kalogeraki,V.S. and Winans,S.C. (1997) Suicide plasmids containing promoterless reporter genes can simultaneously disrupt and create fusions to target genes of diverse bacteria. *Gene*, **188**, 69–75.

5. Metcalf,W.W., Jiang,W., Daniels,L.L., Kim,S.K., Haldimann,A. and Wanner,B.L. (1996) Conditionally replicative and conjugative plasmids carrying *lacZ* alpha for cloning, mutagenesis, and allele replacement in bacteria. *Plasmid*, **35**, 1–13.

6. De Lorenzo,V., Herrero,M., Jakubzik,U. and Timmis,K.N. (1990) Mini-Tn5 transposon derivatives for insertion mutagenesis, promoter probing, and chromosomal insertion of cloned DNA in gram-negative eubacteria. *J. Bacteriol.*, **172**, 6568–72.

7. Grinter,N.J. (1983) A broad-host-range cloning vector transposable to various replicons. *Gene*, **21**, 133–43.

8. Simon,R., Priefer,U. and Pühler,A. (1983) A Broad Host Range Mobilization System for *In Vivo* Genetic Engineering: Transposon Mutagenesis in Gram Negative Bacteria. *Bio/Technology*, **1**, 784–791.

9. Przybilski,R., Richter,C., Gristwood,T., Clulow,J.S., Vercoe,R.B. and Fineran,P.C. (2011) Csy4 is responsible for CRISPR RNA processing in *Pectobacterium atrosepticum*. *RNA Biol.*, **8**, 517–528.

10. Richter,C., Dy,R.L., McKenzie,R.E., Watson,B.N.J., Taylor,C., Chang,J.T., McNeil,M.B., Staals,R.H.J. and Fineran,P.C. (2014) Priming in the Type I-F CRISPR-Cas system triggers strand-independent spacer acquisition, bi-directionally from the primed protospacer. *Nucleic Acids Res.*, 8516-26.

11. Gristwood,T., McNeil,M.B., Clulow,J.S., Salmond,G.P.C. and Fineran,P.C. (2011) PigS and PigP regulate prodigiosin biosynthesis in *Serratia* via differential control of divergent operons, which include predicted transporters of sulfur-containing molecules. *J. Bacteriol.*, **193**, 1076–85.

1. F = Forward Primer [↑](#footnote-ref-1)
2. R = Reverse Primer [↑](#footnote-ref-2)
